# Vibrational Properties in Highly Strained Hexagonal Boron Nitride Bubbles

**DOI:** 10.1021/acs.nanolett.1c04197

**Published:** 2022-02-02

**Authors:** Elena Blundo, Alessandro Surrente, Davide Spirito, Giorgio Pettinari, Tanju Yildirim, Carlos Alvarado Chavarin, Leonetta Baldassarre, Marco Felici, Antonio Polimeni

**Affiliations:** † Physics Department, Sapienza University of Rome, Piazzale Aldo Moro 5, 00185 Rome, Italy; ‡ Department of Experimental Physics, Faculty of Fundamental Problems of Technology, Wroclaw University of Science and Technology, Wroclaw 50-370, Poland; § IHP-Leibniz Institut fur Innovative Mikroelektronik, Im Technologiepark 25, 15236 Frankfurt (Oder), Germany; ∥ Institute for Photonics and Nanotechnologies (CNR-IFN), National Research Council, 00156 Rome, Italy; ⊥ Center for Functional Sensor & Actuator (CFSN), National Institute for Materials Science (NIMS), Tsukuba, Ibaraki 305-0044, Japan

**Keywords:** strain, hBN, 2D materials, Raman, nano-IR, phonons

## Abstract

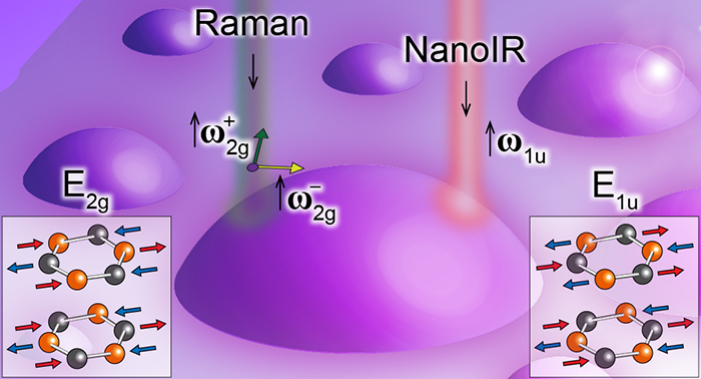

Hexagonal boron nitride (hBN) is widely used as a protective layer for few-atom-thick crystals and heterostructures (HSs), and it hosts quantum emitters working up to room temperature. In both instances, strain is expected to play an important role, either as an unavoidable presence in the HS fabrication or as a tool to tune the quantum emitter electronic properties. Addressing the role of strain and exploiting its tuning potentiality require the development of efficient methods to control it and of reliable tools to quantify it. Here we present a technique based on hydrogen irradiation to induce the formation of wrinkles and bubbles in hBN, resulting in remarkably high strains of ∼2%. By combining infrared (IR) near-field scanning optical microscopy and micro-Raman measurements with numerical calculations, we characterize the response to strain for both IR-active and Raman-active modes, revealing the potential of the vibrational properties of hBN as highly sensitive strain probes.

## I Introduction

Hexagonal boron nitride (hBN), a wide-gap layered material,^1^ features a marked chemical inertness^2,3^ and mechanical robustness.^4^ Thanks to these properties, hBN is an ideal substrate or capping material for two-dimensional crystals,^5−10^ protecting them from oxidation^11^ and bringing about a substantial improvement of the charge-carrier mobility and of the light emission characteristics.^7,8,12^ Indeed, hBN capping is routinely employed to fabricate high-quality heterostructures (HSs), wherein intriguing carrier potential landscapes can be realized.^13,14^ The fabrication process relies on mechanical stacking, often leading to the emergence of strain in the different layers and to important modifications of their electronic states.^15^ hBN is also attracting increasing interest for its intrinsic properties, sustaining the propagation of hyperbolic phonon-polaritons (HPPs)^16,17^ and hosting single-photon emitters operating at room temperature.^18−22^ Its remarkable mechanical robustness (breaking strengths of ∼70 GPa and Young’s modulus of ∼800 GPa^4,23,24^) was exploited for high-quality mechanical resonators^25^ and to reversibly tune the emission wavelength of single-photon emitters via stretching.^26^ Strained wrinkles were also found to be ideal candidates for launching HPPs.^27^ It follows that in hBN, like in other two-dimensional materials, strain plays a relevant role.^24^ Different methods were employed to induce strain in thin layers of hBN, for example, by deposition on substrates subject to stretching,^26^ bending,^28^ or thermal compression^29^ or by nanoindentation.^4^ Great attention was also attracted by the formation of hBN bubbles ensuing gas trapping,^30^ hydrogen-plasma exposure,^31^ or pressure-induced bulging.^23^ Such bubbles may be the ultimate platforms for probing the elastic/adhesive properties of two-dimensional materials, owing to the strong interplay between these properties and the bubble morphology.^23,32−34^ Although hBN bubbles are expected to host sizable strains, as theoretically predicted and experimentally confirmed in similar graphene^35^ and transition-metal dichalcogenide (TMD) structures,^33,36−40^ where total strains of 1–5% were achieved, no clear evidence of strain has been provided so far. More generally, the effect of strain on the vibrational properties of thin hBN has surprisingly not received systematic attention, with only a few Raman studies published to date, focusing on the moderate strain regime (<0.4%).^28,29,41^

Here we report on a method to mechanically deform hBN based on the low-energy hydrogen (H) or deuterium (D) ion irradiation of multilayer flakes. Depending on the flake thickness, H/D-ion treatments lead to the formation of nano/micrometric bubbles or wrinkles. Unlike methods based on the deposition of ultrathin films,^30^ the proposed technique permits the formation of wrinkles and bubbles with a high density and on flakes with virtually unrestricted size. In addition, we can control the thickness of the bubbles from a few layers to tens of layers by tuning the energy or changing the isotope of the ion beam. We employed an infrared (IR) scanning near-field optical microscope (SNOM) to perform nanoscale Fourier transform IR (nano-FTIR) measurements and an optical microscope to perform micro-Raman (μ-Raman) measurements. Across the bubble surfaces, we observe record large shifts of both the IR-active and Raman-active modes in excess of 50 cm^–1^. With the support of numerical modeling of the strain distribution, we extract the Grüneisen parameters of hBN and, by performing linearly polarized Raman spectroscopy, its shear deformation potential.

## II Results and Discussion

**Figure 1:**
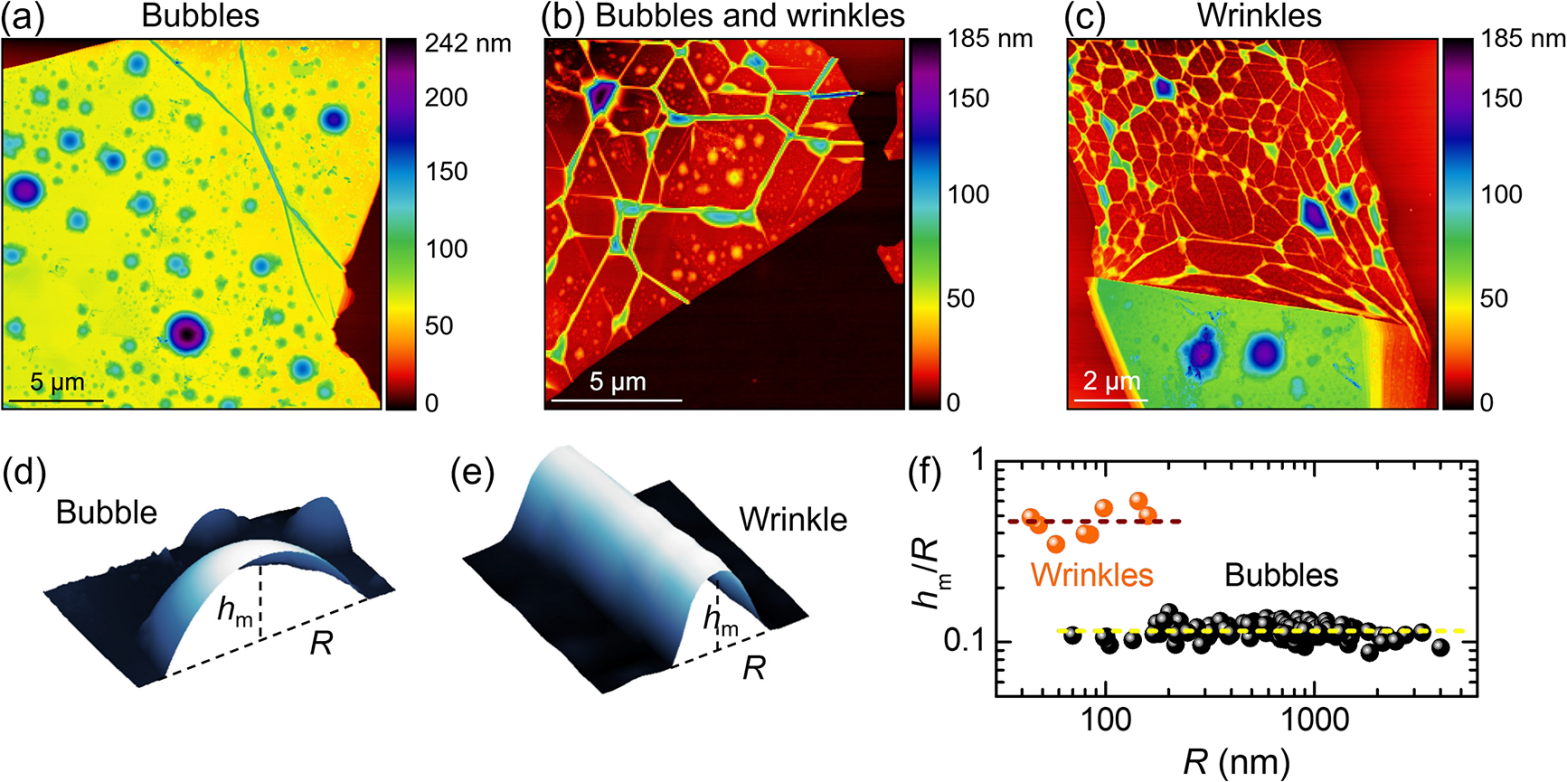
Formation of hydrogen-filled bubbles and of wrinkles in hBN. (a–c) AFM images of multilayer hBN flakes after H irradiation. The flakes have thicknesses of (a) 55, (b) 10, and (c) 5 nm (thin part corresponding to the top side of the figure), and the images show the presence of only bubbles, both bubbles and wrinkles, and only wrinkles, respectively. (d,e) 3D AFM images of half a bubble (panel d, where *R* = 2.06 μm and *h*_m_ = 225 nm) and part of a wrinkle (panel e, where *R* = 144 nm and *h*_m_ = 88 nm). The definitions of maximum height (*h*_m_) and footprint radius (*R*) are depicted. (f) Statistical analysis of the aspect ratios (*h*_m_/*R*) measured in wrinkles and bubbles. The dashed lines represent the average aspect ratios estimated for each set of data.

We exfoliated thick hBN flakes from commercial hBN crystals (HQ graphene). The flakes were deposited on Si/SiO_2_ substrates and initially characterized by atomic force microscopy (AFM); see the Supporting Information, Methods. The samples were subjected to H (or D)-ion irradiation by a Kaufman ion gun^37,42^ under high vacuum conditions at 150 °C, with the samples electrically grounded to avoid charging. For details, see the Supporting Information, Methods. To avoid the formation of defects, we employed low ion-beam energies of <35 eV. After the treatment, optical microscope images of the flakes may reveal a slightly nonhomogeneous coloration related to the presence of barely visible circular or elongated features; see Supporting Figure S1. AFM measurements demonstrate the presence of bubbles, wrinkles, or both on the flakes, as shown in Figure 1a–c and Supporting Figure S2. A statistical AFM study (see Supporting Figure S3) allows us to establish a correspondence between the different morphologies and the flake thickness *t*: For *t* ≳ 10 nm, only bubbles form (Figure 1a); for *t* ≃10 nm, both bubbles and wrinkles can be observed (Figure 1b); and in thin flakes with *t* ≲ 10 nm, wrinkles and irregular structures predominate (Figure 1c). In the latter case, molecular hydrogen likely forms, accumulates, and percolates at the flake/substrate interface, giving rise to irregular structures and wrinkles (Figure 1c); see also Supporting Figure S3. On the contrary, the formation of spherically shaped bubbles in thick flakes (*t* ≳ 10 nm) can be attributed to the formation and trapping of molecular hydrogen in the hBN interlayers, as observed in H-plasma-treated hBN,^31^ and in TMDs.^37^ We thus hypothesize that protons with kinetic energies of ∼10–30 eV penetrate through hBN for ∼10 nm and that the formation of wrinkles or bubbles depends on where H_2_ remains caged. To support this hypothesis, we intentionally induced the explosion of some bubbles via a high-power (some milliwatts), highly focused laser beam and measured the height difference between the crater of the exploded bubble and the flake surface outside the crater by AFM. In samples irradiated with H ions (beam energies <34 eV) (see Supporting Figure S4), we measured thicknesses ranging from 1.8 to 12 nm (corresponding to about 5 to 36 monolayers). To form thinner bubbles, instead, we irradiated some samples with deuterium ions (beam energies <25 eV), which are known to penetrate less through hBN with respect to protons,^43^ and we measured bubble thicknesses as thin as ∼0.5 nm (i.e., a couple of layers); see Supporting Figure S4. This demonstrates the remarkable flexibility of our method, which, unlike H-plasma-based methods,^31^ enables us to obtain bubbles thinner than 10 monolayers. The long durability of the bubbles and Raman studies of the irradiated flakes (see Supporting Figure S5) suggest that the low-energy beams employed here do not induce a sizable amount of defects in the crystal, unlike higher energy heavier atom beams.^44−50^

We performed AFM measurements to study the morphological properties of bubbles and wrinkles and measured their aspect ratio *h*_m_/*R*, where *h*_m_ is the maximum height of the object and *R* is its half width. (See Figure 1d,e). The results are summarized in Figure 1f. The wrinkles feature a narrow width distribution and aspect ratios in the 0.3 to 0.6 range. The bubbles show a much wider size distribution and a size-independent aspect ratio, as expected based on previous theoretical^30,32,51^ and experimental^30−32,37,38,52^ studies. For our bubbles, we find *h*_m_/*R* = 0.115 ± 0.011, in agreement with that reported for hydrocarbon-filled monolayer bubbles^30^ and multilayer bubbles created by H-plasma treatments.^31^ The constant aspect ratio, independent of size and thickness, testifies that the mechanics of the bubbles is dominated by stretching, whereas the bending contribution is negligible,^32,53^ at variance with other kinds of bent, yet not pressurized, systems.^54^ Importantly, the strain scales as (*h*_m_/*R*)^2^;^32^ therefore, a similar strain distribution is expected independent of the bubble formation method and thickness. Next, we address such distribution on hBN bubbles.

**Figure 2:**
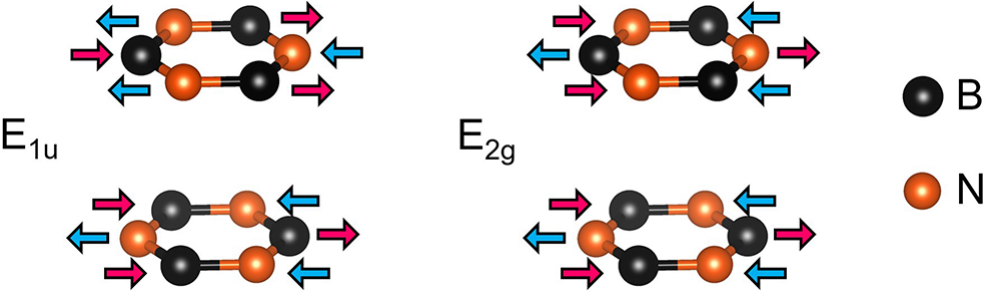
Sketch of the atom displacements corresponding to the IR-active E_1u_ mode and to the Raman-active E_2*g*_ mode. Differently colored arrows indicate opposite atom motions.

One of the most common means for evaluating the amount of strain in two-dimensional materials is provided by a quantitative analysis of the frequency of the lattice vibration normal modes.^24^ Typically, lattice stretching (i.e., tensile strain) induces a softening of the phonon modes. Furthermore, under anisotropic strains, the double-degenerate in-plane modes split as a result of the lowered crystal symmetry. The shift rate and splitting rate of the vibrational modes can thus be conveniently used to assess the strain magnitude and its anisotropy degree in atomically thin membranes.^24^ This is especially important when the actual strain differs from the expected strain, like in many bending or stretching devices,^24^ or cannot be estimated theoretically. In this work, we focus on two specific in-plane transverse modes, which are IR-active (E_1u_) and Raman-active (E_2g_). Their lattice displacements are sketched in Figure 2.

**Figure 3:**
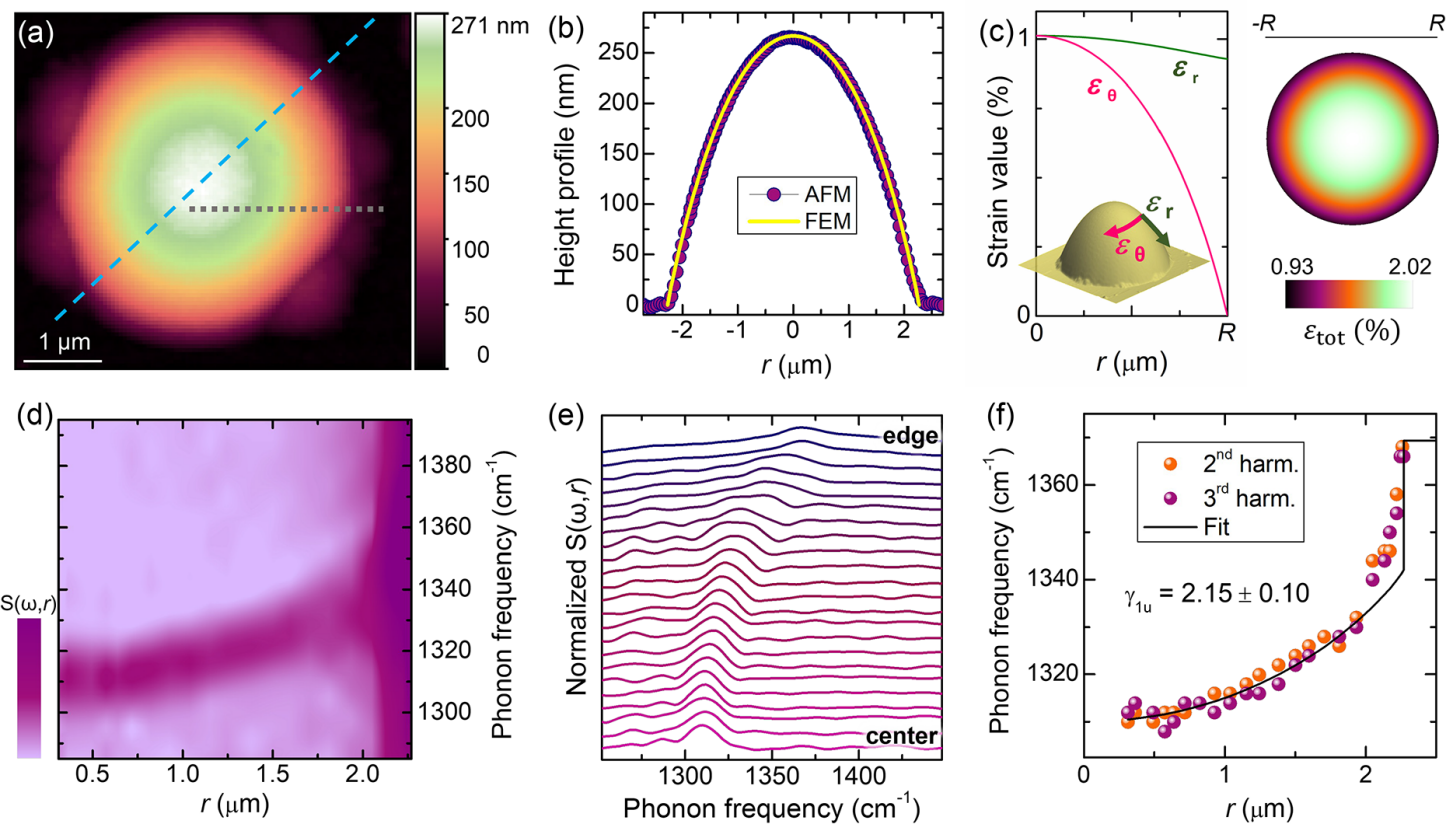
IR-active mode versus strain. (a) 2D AFM image of a hBN bubble exhibiting a circular symmetric shape but on its edge, where smaller satellite bubbles nucleated. The bubble has *R* = 2.27 μm and *h*_m_ = 267 nm (*h*_m_/*R* = 0.117) and was created in a deuterated sample (beam energy equal to 6 eV) to minimize the bubble thickness. (b) Comparison between the AFM profile acquired along a diameter of the bubble (highlighted in panel a by a cyan dashed line) and the profile obtained by FEM calculations. (c) Left: Radial dependence, obtained by FEM calculations, of the in-plane circumferential (ε_θ_) and radial (ε_r_) strain components, a sketch of which is depicted as the inset. Right: Spatial distribution of the total in-plane strain ε_tot_ = ε_r_ + ε_θ_. (d,e) Color map of the near-field amplitude *S*(ω,*r*) (d) and corresponding spectra (e), where the IR-active mode (E_1u_) is visible. The measurements were taken along the gray short dashed line shown in panel a. The second harmonic is considered here. (f) IR phonon frequency dependence on the radial distance *r*, as deduced from the spectra shown in panel e and the AFM profile. The third-harmonic data are also included here. The black solid line is a fit to the data assuming a linear dependence of the phonon frequency on ε_tot_, provided by eqs 3 and 5.

Figure 3a displays the AFM image of a circular hBN bubble with diameter *D* = 2*R* = 4.54 μm and height *h*_m_ = 267 nm (*h*_m_/*R* = 0.117) obtained by D irradiation. The AFM profile recorded along the cyan dashed line is shown in Figure 3b (circles). The yellow line is the profile evaluated by finite element method (FEM) numerical calculations; see the Supporting Information, Methods. The latter also provides the strain distribution,^32,37^ as shown on the left side of Figure 3c, where ε_r_ and ε_θ_ are the radial and circumferential in-plane strain components in polar coordinates, respectively.^32,53^ The calculated spatial distribution of the total strain ε_tot_ = ε_r_ + ε_θ_ is displayed as a false-color image on the right side of panel c. Strain features an anisotropic character, changing from tensile uniaxial at its edge (*r*/*R* = 1, ε_r_ ≠ 0 and ε_θ_ = 0) to tensile equi-biaxial at the summit of the bubble (*r*/*R* = 0, ε_r_ = ε_θ_). On these premises, we expect the in-plane transverse phonon frequency ω_*t*_ to undergo a decrease with respect to unstrained hBN due to stretching, as well as a splitting in ω_*t*_^+^ and ω_*t*_^–^, the extent of which depends on the position on the bubble. Thus we introduce the average frequency
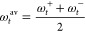
1and mode splitting
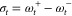
2The frequency variation upon strain can be quantified by the shift rate
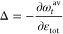
3and splitting rate
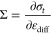
4where ε_tot_(*r*) = ε_r_(*r*) + ε_θ_(*r*) and ε_diff_(*r*) = ε_r_(*r*) – ε_θ_(*r*).

Equivalently, one can introduce dimensionless quantities, such as the Grüneisen parameter
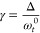
5and the shear deformation potential
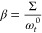
6where ω_*t*_^0^ is the mode frequency in the absence of strain.

The E_1u_ mode (see lattice displacements in Figure 2) was studied by nano-FTIR SNOM measurements;^55,56^ see the Supporting Information, Methods. This technique has been widely employed in two-dimensional systems, for example, to probe phonon-polaritons in hBN,^27,57,58^ phonons in hBN superlattices,^59^ electron–phonon interactions in graphene,^60^ and intersubband transitions in two-dimensional quantum wells,^61^ but the E_1u_ hBN mode sensitivity to strain has not been investigated, to our knowledge. Figure 3d shows the normalized near-field amplitude *S*(ω,*r*), as obtained with a spectral line scan along the gray short dashed line in Figure 3a. The near-field signal originates from the tip–sample interaction and provides a lateral resolution of ∼20 nm; see the Supporting Information, Methods. The corresponding spectra are shown in Figure 3e. The phonon peak frequency from the bulk region outside the bubble is ω_1u_ = 1367 cm^–1^, in agreement with previous reports.^62^ An abrupt decrease in ω_1u_ is noticed when the tip approaches the bubble’s edge, where a 0.9% tensile strain is already present. (See Figure 3c.) On moving further toward the bubble center, ω_1u_ seamlessly decreases, in agreement with the expected tensile strain increase. To quantify the mode shift variation versus the total strain ε_tot_(*r*), we established a one-to-one correspondence between the AFM-derived bubble profile (*h* vs *r*) and the calculated strain components shown in Figure 3c. In turn, this allowed us to establish a correspondence between each measured ω_1u_ and ε_tot_(*r*), given that the *h*(*r*) values were measured by the SNOM tip at the very same points where ω_1u_ was probed. To reduce the background signal, we collected the near-field data at several harmonics. In Figure 3f, we show the spatial dependence of the second and third harmonics of the signal associated with the E_1u_ phonon. (See the Supporting Information, Methods and Supporting Note 1.) We reproduce quite successfully the dependence of ω_1u_ on *r* using as fitting parameters the mode frequency at zero strain ω_1u_^0^ = (1369.7 ± 2.4) cm^–1^ and the shift rate Δ_1u_ = (29.5 ± 1.4) cm^–1^/%, resulting in a Grüneisen parameter (see eq 5) γ_1u_ = 2.15 ± 0.10. Analogous measurements were performed on other bubbles; see Supporting Note 1 and Table 1.

**Table 1:** Effect of Strain on the Vibrational Modes^a^

mode	ω_*t*_^0^ (cm^–1^)	Δ (cm^–1^/%)	γ_*t*_	Σ_*t*_ (cm^–1^/%)	β_*t*_	β_*t*_/γ_*t*_
E_1u_ (IR)	1369.9 ± 2.3	29.4 ± 1.8	2.15 ± 0.12			
	1369.7 ± 2.4	29.5 ± 1.4	2.15 ± 0.10			
	1369.0 ± 5.2	36.2 ± 3.6	2.64 ± 0.27			
E_2g_ (Raman)	1370^b^	24.6 ± 0.60	1.79 ± 0.04	11.2 ± 1.9	0.82 ± 0.14	0.46 ± 0.08
	1370^b^	25.1 ± 4.5	1.83 ± 0.33			
	1370^b^	28.5 ± 8.4	2.08 ± 0.61	15.6 ± 3.8	1.14 ± 0.28	0.56 ± 0.14
	1370^b^	33.2 ± 5.2	2.43 ± 0.40			

a Parameters obtained for the E_1u_ and E_2g_ from the nano-FTIR and Raman measurements, respectively, The frequency at zero strain (ω_*t*_ ), shift rate (Δ), Grüneisen parameter (γ_*t*_ ), splitting rate (Σ_*t*_ ), shear deformation potential (β_*t*_ ), and ratio γ_*t*_/β_*t*_ were estimated for several bubbles.

b This value was kept fixed because it was otherwise affected by too large uncertainties.

It should be noticed that the zero-strain limit ω_1u_^0^ (∼1370 cm^–1^) of the bubble E_1u_ mode is larger than that of bulk hBN (∼1367 cm^–1^). This is consistent with the frequency increase reported for the Raman-active E_2g_ mode in the few-layer limit.^29,63,64^

**Figure 4:**
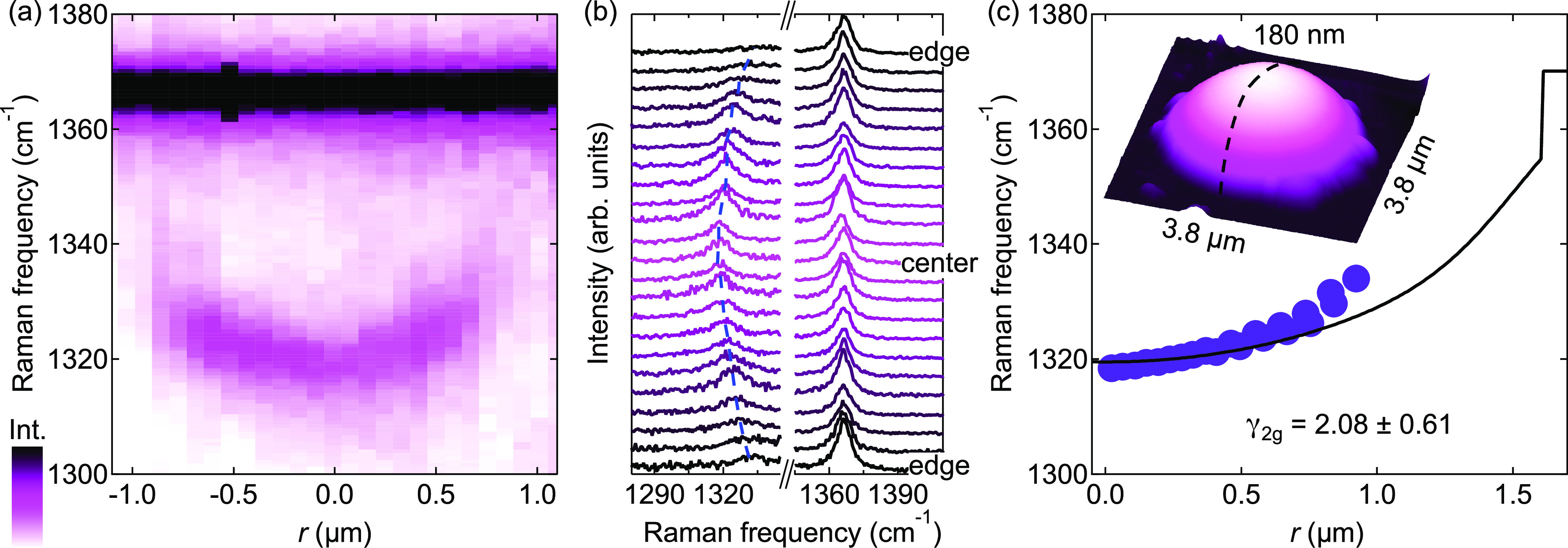
Raman-active mode versus strain. (a) False-color image of the intensity of the E_2g_ Raman mode as a function of the position along a diameter of a bubble. The bubble has *R* = 1.61 μm and *h*_m_ = 179 nm (*h*_m_/*R* = 0.111) and was created in a deuterated sample (beam energy equal to 25 eV). (b) Raman spectra corresponding to the map of panel a. (c) E_2g_ Raman-mode frequencies as a function of the distance from the center of the bubble. The solid line is a linear fit to the frequency versus *r* behavior, with γ_2g_ being the fitting parameter. Inset: AFM image of the investigated structure. The dashed line indicates the diameter along which the spectra were measured.

Let us now discuss our studies of the E_2g_ mode. (See the lattice displacements in Figure 2.) We performed μ-Raman measurements of the hBN bubble (*R* = 1.61 μm, *h*_m_ = 179 nm, *h*_m_/R = 0.111, created by D irradiation), whose AFM image is shown as the inset of Figure 4c. Figure 4a is the spectrally and spatially resolved intensity map of the light scattered by the bubble in the spectral region of the E_2g_ mode. The map was recorded along a diameter (see the inset of panel c), and the corresponding spectra are shown in Figure 4b. The spot size and spectral resolution are ∼0.5 μm and 0.7 cm^–1^, respectively; see the Supporting Information, Methods. The intense peak at 1366.2 cm^–1^ comes from the bulk hBN flake from which the bubble swelled. The E_2g_ signal from the bubble is much less intense due to the reduced thickness and exhibits a spatially dependent and lower frequency due to strain. We notice that unlike the IR signal, the Raman signal becomes negligibly small as the laser approaches the edge of the bubble due to optical interference effects.^37,39^ The correspondence between the measured ω_2g_ values and ε_tot_(*r*) = ε_r_(*r*) + ε_θ_(*r*) is established by evaluating the strain via FEM calculations based on the AFM profile; see Supporting Figure S6. The spatial dependence of ω_2g_ is shown in Figure 4c, and it is best reproduced with a shift rate Δ_2g_ = (28.5 ± 8.4) cm^–1^/% and a Grüneisen parameter (see eq 5) γ_2g_ = 2.08 ± 0.61. The extrapolation frequency at zero strain was set at 1370 cm^–1^, which is greater than the corresponding bulk mode (1366.2 cm^–1^), like in the case of the E_1u_ IR-active mode and consistent with published results.^29,63,64^ Similar measurements performed on different bubbles are shown in Supporting Note 2, and the estimated parameters are displayed in Table 1. We also performed a statistical analysis of the shift at the bubble summit including many other bubbles, giving average Grüneisen parameters γ_2g_ = 2.04 ± 0.48 (Δ_2g_ = (27.9 ± 6.6) cm^–1^/%); see Supporting Note 3. Our statistical analysis also shows how E_1u_ and E_2g_ are characterized by similar Grüneisen parameters.

Previous μ-Raman studies on hBN bubbles created by H-plasma treatments^31^ reported only a modest shift of ∼3 cm^–1^ between the bubble center and the bulk hBN. Similar small shifts (∼3 cm^–1^) were observed in hBN monolayers subject to thermal compression (biaxial strain of −0.17%),^29^ resulting in γ_2g_ = 0.62. Finally, uniaxial strains of up to 0.4% were applied to thin hBN flakes (two to four layers) using a bending apparatus, achieving frequency softenings of <6 cm^–1^. Grüneisen parameters γ_2g_ between 1.77 and 2.07 were estimated in this case^28^ and were, on average, slightly lower than our estimates. (See Table 1.) By comparison with the current literature, our approach permits us to achieve a much larger total strain, on average, equal to ∼1.9%, with unprecedented shifts in excess of 50 cm^–1^.

**Figure 5:**
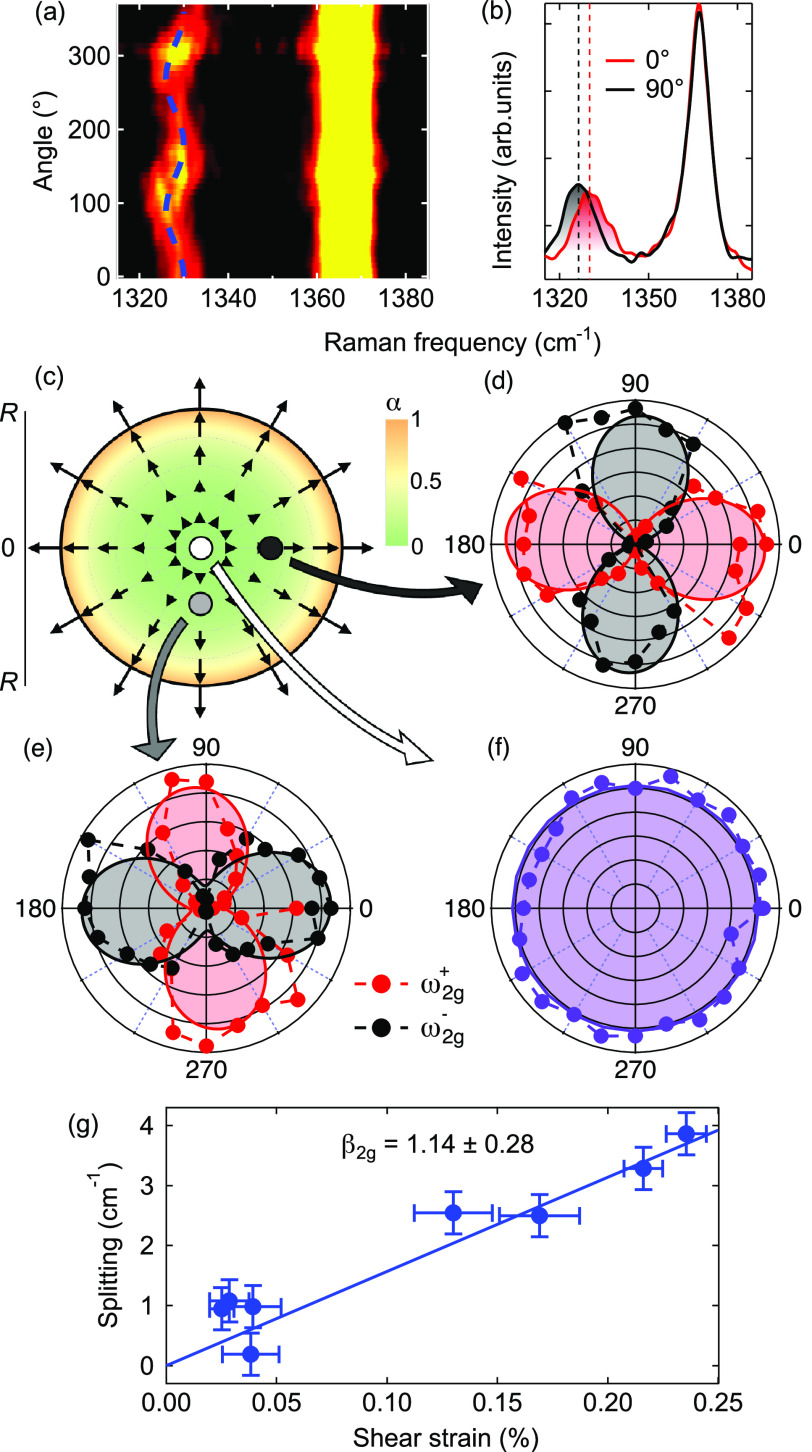
(a) False-color map of the intensity of the E_2g_ Raman mode as a function of the angle of the polarization analyzer. The dashed line is a sinusoidal guide to the eye. (b) μ-Raman spectra measured with polarizations parallel and perpendicular to the uniaxial strain direction. (c) Radial dependence of the strain anisotropy α = (ε_r_ – ε_θ_)/(ε_r_ + ε_θ_), based on FEM calculations. The arrows point to the direction of the strain field. Their length is calculated as log_10_(100α). The dots depict the position of the excitation spots of the polarization-resolved Raman measurements. (d–f) Intensity of the low-frequency (ω_1u_^–^) and high-frequency (ω_1u_^+^) Raman modes as a function of the analyzer angle for excitation performed (d) on the right (black dot), (e) at the bottom (gray dot), and (f) at the center of a bubble (white dot). (g) Mode splitting as a function of the shear strain. The solid line is a linear fit.

In addition to the E_2g_ mode shift, a splitting is expected in the bubbles due to the imbalance between ε_θ_ and ε_r_; see Supporting Figure S6. Figure 5a displays an intensity map formed by polarization-dependent μ-Raman spectra recorded on a given point of the same bubble of Figure 4. The point is 790 nm away from the center (i.e., *r*/*R* = 0.49) and is marked by a black dot superimposed on the strain anisotropy degree plot in Figure 5c, with the anisotropy being defined as α = (ε_r_ – ε_θ_)/(ε_r_ + ε_θ_). Therein, the arrows indicate the strain direction. The radial distance *r* was determined by the relationship between ω_2g_ and *r* given in Figure 4c. Each spectrum of Figure 5a was recorded by keeping the polarization direction of the laser fixed at an arbitrary, unknown angle ϕ_0_ with respect to a reference crystal direction (e.g., the armchair/zigzag direction). Likewise, strain is oriented along the bubble radius, and its direction is thus also fixed at an unknown angle θ with respect to the same lattice reference. The angle ϕ between the polarization of the Raman-scattered and Raman-exciting photons was then varied from 0 to 360°. Whereas the E_2g_ bulk mode at 1366.2 cm^–1^ remains constant in intensity and frequency, the strain-softened E_2g_ mode of the bubble in the 1320–1340 cm^–1^ range exhibits a marked angular dependence of its center-of-mass frequency, pointing to a mode splitting. This is exemplified in Figure 5b, showing two μ-Raman spectra recorded with opposite polarizations (ϕ = 0 and 90°). Indeed, it can be demonstrated that the intensities I_2g_^±^ of the E_2g_^±^ modes split by uniaxial strain are given by^24^
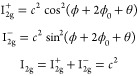
7where *c* is a constant. By performing a line-shape fitting of the Raman spectra (see Supporting Note 4), we extracted I_2g_^±^ as a function of ϕ, where E_2g_^+^ and E_2g_^–^ refer to the high- and low-frequency components, respectively. Figure 5d shows the resulting polar plot obtained from the data of panel a. The reference angle (2ϕ_0_ + θ) is set to zero for simplicity reasons. The two components are clearly in counter phase, as expected. Figure 5e shows a similar set of measurements acquired on a point of the bubble positioned symmetrically at 90° with respect to the previous one (at *r* = 680 nm); see the gray dot in panel c. In this case, the strain direction is given by θ′ = θ + 90°, and as a consequence of eq 7, the E_2g_^±^ components follow an angular dependence that is π/2 out-of-phase with respect to that of the previous point (Figure 5d). These results are fully consistent with the strain field calculated numerically, whereby the ε_r_ component dictates the strain direction. Finally, the μ-Raman spectra recorded at the bubble center (white dot in panel c), where the strain is equi-biaxial, show no mode splitting; see Figure 5f. Other polarization maps were acquired in different points of the bubble. For each point, the average frequency ω_2g_^av^ corresponds to a given *r* value. (See Figure 4c.) In turn, via numerical simulations (see Supporting Figure S6), we obtain ε_shear_(*r*) = ε_diff_(*r*) = ε_r_(*r*) – ε_θ_(*r*). Figure 5g shows the dependence of the mode splitting σ_2g_ versus ε_shear_(*r*). Considering eq 6, we estimate a splitting rate Σ_2g_ = 15.6 ± 3.8 cm^–1^/% and a shear deformation potential β_2g_ = 1.14 ± 0.28. Thus for this bubble, we get β_2g_/γ_2g_ = 0.56 ± 0.14. We performed similar measurements on another bubble with a lower Grüneisen parameter (see Supporting Note 4) and found β_2g_ = 0.82 ± 0.14 and β_2g_/γ_2g_ = 0.46 ± 0.08 (see Table 1), showing how the ratio β_2g_/γ_2g_ is less affected by fluctuations than β_2g_ and γ_2g_. We are aware of only one previous report of the hBN shear potential in the few-layer limit, where the ratio β_2g_/γ_2g_ was found to vary between 0.45 and 0.52.^28^

## III Conclusions

We irradiated bulk hBN flakes with low-energy hydrogen or deuterium ions. The ions penetrate through the crystal for a few nanometers, and molecular hydrogen or deuterium forms, inducing the blistering of a few atomic planes and hence the formation of micro/nano-metric wrinkles or bubbles. Wrinkles or bubbles predominate for flake thicknesses of *t* ≲ 10 nm or ≳10 nm, respectively. The bubbles were investigated in detail because they exhibit tensile strains with a remarkably high ∼2% maximum value, exceeding that typically achieved for hBN in bending/stretching devices.^28,29,41^ The effects of strain on the IR-active (E_1u_) and Raman-active (E_2g_) in-plane modes were studied over the bubble surface by spatially resolved nano-FTIR and polarization-dependent μ-Raman, respectively. The large amount of strain and its anisotropic character toward the edge of the bubbles permitted to derive shift and splitting rates on the order of 30 and 15 cm^–1^/%, respectively. These values are comparable to those reported in graphene and are about one order of magnitude larger than those found in TMDs, InSe, and black phosphorus.^24^ These findings show that the vibrational properties of hBN are extremely sensitive probes of mechanical deformations, and thus they can be exploited to assess the stress status of two-dimensional HSs and hBN-based quantum emitters.

## Supporting Information Available

Methods section and notes on IR-SNOM and Raman mapping or statistical measurements and of polarization-resolved Raman measurements in hBN bubbles, additional optical and AFM images of hBN bubbles and wrinkles, and additional FEM calculations (PDF)
