# Supplementary material for: Vibrational Properties in Highly Strained Hexagonal Boron Nitride Bubbles
Source: Nano Lett. 2022 Feb 2;22(4):1525–33. doi: 10.1021/acs.nanolett.1c04197 (PMC8880391; doi:10.1021/acs.nanolett.1c04197)
Supplement: Supplementary file 1 — nl1c04197_si_001.pdf [file nl1c04197_si_001.pdf]

SUPPORTING INFORMATION for

Vibrational properties in highly strained hexagonal boron nitride bubbles

Elena Blundo,[1] Alessandro Surrente,[1,2] Davide Spirito,[3] Giorgio Pettinari,[4] Tanju Yildirim,[5] Carlos Alvarado Chavarin,[3] Leonetta Baldassarre,[1,3] Marco Felici,[1] and Antonio Polimeni[1,*]

[1] *Physics Department, Sapienza University of Rome, 00185, Roma, Italy.*
[2] *Department of Experimental Physics, Faculty of Fundamental Problems of Technology, Wroclaw University of Science and Technology, Wroclaw 50-370, Poland* [3] *IHP-Leibniz Institut fur Innovative Mikroelektronik, Im Technologiepark 25, 15236 Frankfurt (Oder), Germany*
[4] *Institute for Photonics and Nanotechnologies (CNR-IFN), National Research Council, 00156 Roma, Italy.*
[5] *Center for Functional Sensor and Actuator (CFSN), Research Center for Functional Materials, National Institute for Materials Science (NIMS), 1-1 Namiki, Tsukuba, Ibaraki 305-0044, Japan.*

* Corresponding author: antonio.polimeni@uniroma1.it

Contents

Methods **1**

Supporting Note 1. IR-SNOM mapping measurements in hBN bubbles **3**

Supporting Note 2. Raman mapping measurements in hBN bubbles **6**

Supporting Note 3. Statistical analysis of the shift rates and Grüneisen parameters **7**

Supporting Note 4. Polarization-resolved Raman measurements in hBN bubbles **8**

Supporting Figure 1. Optical and AFM Images of an H-irradiated flake **11**

Supporting Figure 2. AFM characterization before and after H-irradiation **12**

Supporting Figure 3. Bubbles and wrinkles in hBN **13**

Supporting Figure 4. Bubble thickness **15**

Durability of hBN bubbles **16**

Supporting Figure 5. AFM and FEM calculations of the bubble studied by Raman spectroscopy **17**

References **18**

Methods

Sample fabrication and cleaning procedure. hBN flakes were mechanically exfoliated by the scotch tape method and deposited on Si/SiO_2 substrates. AFM measurements were thus performed to check the status of the flake surface before H-ion irradiation. Whenever adhesive tape residuals were found, the samples were cleaned by acetone and IPA baths.

Atomic force microscopy. AFM measurements were performed using a Veeco Digital Instruments Dimension D3100 microscope equipped with a Nanoscope IIIa controller, employing Tapping Mode monolithic silicon probes with a nominal tip curvature radius of $5 - 10\,\mathrm{nm}$ and a force constant of $40\,\mathrm{N\,m^{-1}}$ or metal-coated nano-FTIR tips from NeaSpec. All the scans were performed at room temperature and at ambient conditions. All the data were analysed with the Gwyddion software.

Finite-Element Method calculations. Finite-element method (FEM) calculations using nonlinear-membrane theory were employed to model the profile and strain field of the bubbles [1]. To this end, we employed a model implemented in COMSOL Multiphysics 5.1. Thanks to the mirrored symmetry about the longitudinal axis of the bubbles, an axisymmetric formulation using polar coordinates was used. A line element is used to simulate the membrane with the starting thickness t of a single or several layers of hBN. One end of the line element is subject to a fixed constraint (*i.e.*, null displacement) while the rest of the line is free to move in the longitudinal direction. The presence of a fluid within the bubble is modelled as a pressure load acting on the flat membrane surface, thus causing the bubble inflation. The membrane is deformed until the footprint radius R and the maximum height h_0 reach the target value, which is achieved by varying the internal pressure. The effects of the bending stiffness are neglected since $h_0/t \gg 1.5$ [1]. An extra fine mesh is used for all simulations and a 0.001 convergence setting is used to ensure the numerical accuracy of the solutions. A constant Newtonian solver is used for the numerical solver procedure. Due to the anisotropic stiffness of the layered compounds, an anisotropic stiffness matrix was implemented in the linear elastic material node. The elastic matrix (that relates the stress tensor to the strain tensor) used in this work is [2]:

$$C = \begin{pmatrix} 860.2 & 191.9 & 1.2 & 0 & 0 & 0 \\ 191.9 & 860.2 & 1.2 & 0 & 0 & 0 \\ 1.2 & 1.2 & 5.9 & 0 & 0 & 0 \\ 0 & 0 & 0 & 2.4 & 0 & 0 \\ 0 & 0 & 0 & 0 & 2.4 & 0 \\ 0 & 0 & 0 & 0 & 0 & 334.2 \end{pmatrix}; \tag{0.1}$$

As expected, no differences were observed in the strain components for layer thicknesses from 1 to 20 layers (only very small discrepancies of the order of 10^{-4} were observed).

nano-FTIR measurements. The infrared spectra were collected with a SNOM microscope (NeaSNOM from NeaSpec). A difference-frequency generation laser, yielding a linearly polarized, broadband IR radiation, is focused on a gold-coated AFM probe tip through a parabolic mirror, also used to collect the backscattered radiation. The AFM is operated in tapping mode at $\sim 220\,\mathrm{kHz}$. The scattered signal is demodulated at several higher harmonics n, and, by selecting those with $n \geq 2$, one has a signal dominated by the near-field interaction of the tip with the sample over the far-field scattered background. The SNOM setup is based on an asymmetric Michelson interferometer (the tip and sample are in one of the arms of a Michelson interferometer), which provides both amplitude and phase of the backscattered radiation [3]. The scattered signal is collected by a N_2-cooled MCT (Mercury-Cadmium-Telluride) detector. Measurements were taken by averaging ten interferograms with an $8\,\mathrm{cm^{-1}}$ spectral resolution

[4]. The demodulated phase and amplitude signals are normalized to the reference quantities measured on an Au patches in the vicinity of the flakes of interest as

$$\eta_n(\omega) = s_n^{\mathrm{norm}} \mathrm{e}^{\mathrm{i}\phi_n^{\mathrm{norm}}} = \frac{s_n^{\mathrm{flake}}}{s_n^{\mathrm{ref}}} \mathrm{e}^{\mathrm{i}\left(\phi_n^{\mathrm{flake}} - \phi_n^{\mathrm{ref}}\right)}, \tag{0.2}$$

where s is the near-field amplitude, ϕ the phase, and the suffix n indicates the nth harmonic demodulation. As shown in the literature for materials such as hBN, where one measures strong phonon modes, the scattered amplitude provides the best way to identify the vibrational modes of the flake [5, 6]. It should be noticed that for the SNOM technique, polarization plays a relevant role in many cases, such as the intersubband transitions in two-dimensional heterostructures, where the transition can only be excited by light polarized in the vertical direction [7]. For hBN phonons, however, the SNOM technique allows one to measure both the in-plane and out-of plane transverse optical modes [5, 8].

Raman measurements. For Raman measurements, the excitation laser was provided by a single frequency Nd:YVO_4 lasers (DPSS series by Lasos) emitting at 532 nm, or by a diode laser emitting at 405 nm. The Raman signal was spectrally dispersed by a 750 mm focal length ACTON SP750 monochromator equipped with a 1200 groove/mm grating and detected by a back-illuminated N_2-cooled Si CCD camera (100BRX by Princeton Instruments). The laser light was filtered out by a very sharp long-pass Razor edge filter (Semrock). The micro-Raman (μ-Raman) spectral resolution was $0.7\,\mathrm{cm}^{-1}$. A 100× objective with NA=0.9 was employed to excite and collect the light, in a backscattering configuration. The laser spot size was experimentally determined as follows: The laser was scanned across a reference sample, lithographically patterned with features of known width (1 µm). The intensity of the reflected light was fitted with the ideal reflectance profile, convolved with a Gaussian peak. The standard deviation of this peak, obtained as a fitting parameter, provides our estimate of $\sigma = 0.23 \pm 0.01\,\mu\mathrm{m}$. In establishing the correspondence between strain and radial distance r, a convolution of the strain distribution within the laser spot was duly taken into account.

Supporting Note 1. IR-SNOM mapping measurements in hBN bubbles

Comparison between different harmonics

nanoFTIR spectra are measured with a near-field microscope (sSNOM, NeaSNOM from Nea-SPEC, see Methods). The broadband illumination is focused via a parabolic mirror on the sample and the AFM tip. As the focus is diffraction-limited, there is an unavoidable background that arises from the light scattered by the sample regions that are not under the tip apex and by the tip shaft. In order to suppress the contribution of the background to the total signal, it is possible to demodulate the signal at high harmonics of the tip oscillation frequency, thus obtaining the near-field signal scattered from the volume under the tip only [9]. In Fig. 1.1, we show line scans taken on the bubble of Fig. 3 of the main text, ad specifically along the grey short-dashed line in Fig. 3(a). Supporting Fig. 1.1 compares the signal amplitude $S(\omega, r)$ at different harmonic demodulations. The scattered signal recorded at the fundamental harmonic shows the signature of the phonon peak of the unstrained hBN coming from outside the bubble or from beneath the bubble together with the red-shifted phonon mode of the strained material. Moving to the amplitude demodulated at the second harmonic, we can detect only the signal scattered by the thin strained hBN that comprises the bubble, and an almost identical line scan is obtained at the 3rd harmonic demodulation.

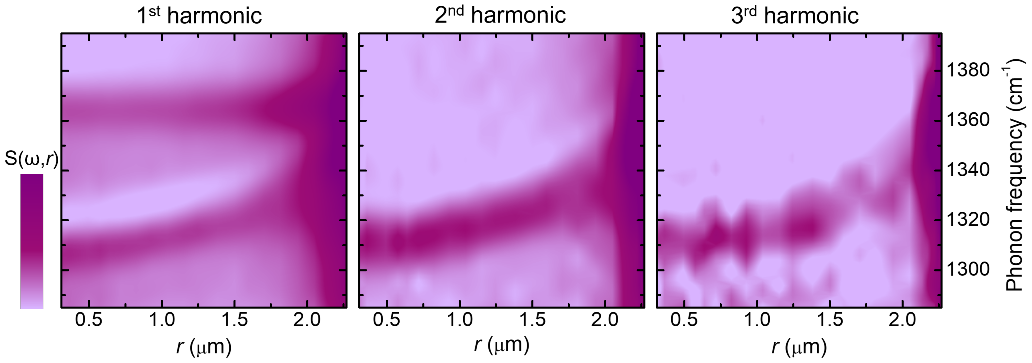

Figure 1.1: Comparison among the first three harmonics of the scattered signal obtained by nanoFTIR line scans taken along the grey short-dashed line in Fig. 3(a) of the main text.

Mapping measurements of other bubbles

To complement the results presented in Fig. 3 of the main text, we performed similar nanoFTIR measurements on other two bubbles. In Fig. 1.2(a), we show the AFM image of the second bubble we studied. This bubble has $R = 0.94$ μm and $h_{\mathrm{m}} = 99$ nm, resulting in $h_{\mathrm{m}}/R = 0.105$. The height profile of this bubble measured along a diameter (cyan dashed line in panel (a)) is shown in panel (b). The nanoFTIR measurements were taken along the same diameter. FEM calculations reproduce quite well the height profile. Indeed, this bubble features a lower aspect ratio with respect to that of Fig. 3 of the main text. This results in a lower strain, whose maximum value reaches 1.5 %, as shown in panel (c). Analogously to the case of the previous bubble, we show the first three harmonics of the scattered amplitude associated to the IR phonon, see panel (d). The measurements were performed along the cyan dashed line superimposed to the AFM image in panel (a). Similarly to the previsou bubble, the 1st harmonic shows the signature of both the strained bubble phonon peak and that of the unstrained hBN outside or

beneath the bubble. In the second harmonic there is still a weak trace of the unstrained hBN, which is instead negligible in the line scan of the third harmonic. By exploiting the one-to-one correspondence between $\varepsilon_{\mathrm{tot}} = \varepsilon_r + \varepsilon_\theta$ and r determined in panel (c), we performed a fit to the data in panel (e), providing an extrapolation frequency at null strain $\omega^0_{\mathrm{1u}} = (1369.0 \pm 5.2)$ cm^{-1} and a shift rate $\Delta_{\mathrm{1u}} = (36.2 \pm 3.6)$ cm^{-1}/%, resulting in a Grüneisen parameter (see Equation 5 of the main text) $\gamma_{\mathrm{1u}} = 2.64 \pm 0.27$.

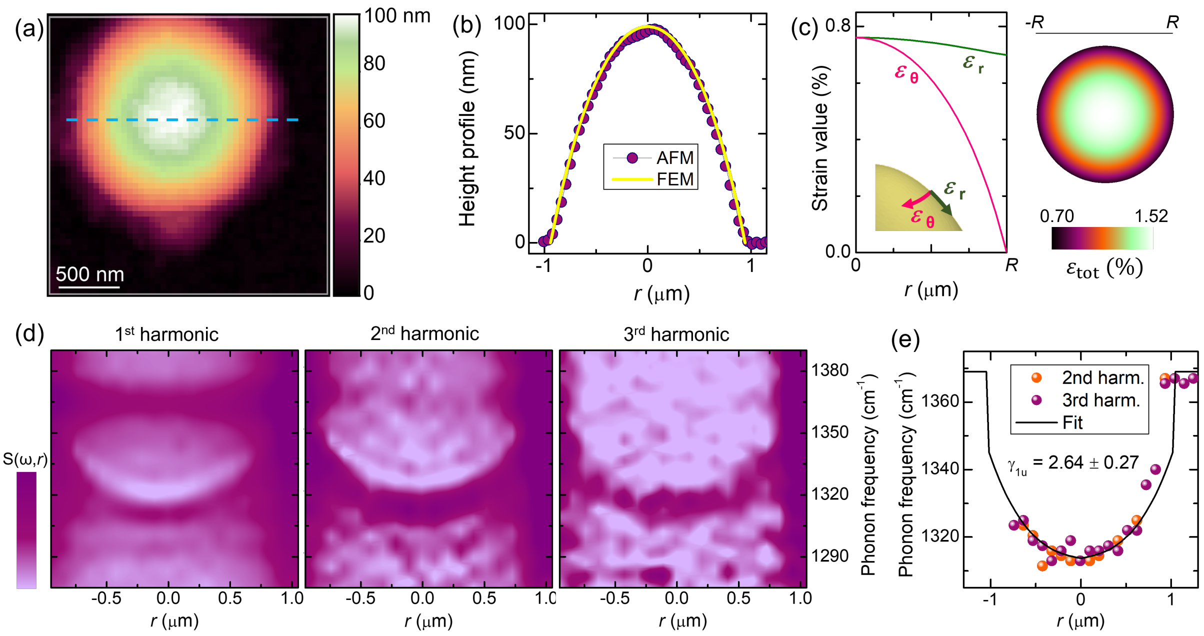

Figure 1.2: IR-active mode *vs* strain for a second bubble. (a) 2D AFM image of a circular hBN bubble, with $R = 0.94$ μm and $h_{\mathrm{m}} = 99$ nm ($h_{\mathrm{m}}/R = 0.105$) and created in a deuterated sample (beam energy equal to 6 eV). (b) Comparison between the AFM profile acquired along a diameter of the bubble (highlighted in panel (a) by a cyan dashed line) and the profile obtained by FEM calculations. (c) Left: Radial dependence —obtained by FEM calculations— of the in-plane circumferential (ε_θ) and radial (ε_r) strain components, a sketch of which is depicted as inset. Right: Spatial distribution of the total in-plane strain $\varepsilon_{tot} = \varepsilon_r + \varepsilon_\theta$. (d) Color map of the intensity of the first three harmonics of the signal associated to the IR active mode (E_{1u}) while scanning the SNOM tip along the cyan dashed line shown in panel (a). (e) IR phonon frequency dependence of the 2nd and 3rd harmonic on the radial distance r. The black solid line is a fit to the data assuming a linear dependence of the phonon frequency on $\varepsilon_{\mathrm{tot}}$, provided by Eqs. (3) and (5) of the main text.

Analogous measurements were performed for a third bubble, whose AFM mage is shown in Fig. 1.3(a). In this case, the linescan was acquired along a radius to limit the map acquisition time. The corresponding color plot is given in panel (b). A similar analysis to that discussed for the previous two bubbles was performed also for this bubble. As summarized in panel (c), from the frequency dependence on r —and in turn on $\varepsilon_{\mathrm{tot}} = \varepsilon_r + \varepsilon_\theta$— we determined a strain-free frequency $\omega^0_{\mathrm{1u}} = (1369.9 \pm 2.3)$ cm^{-1} and the shift rate $\Delta_{\mathrm{1u}} = (29.4 \pm 1.8)$ cm^{-1}/%, resulting in a Grüneisen parameter (see Equation 5 of the main text) $\gamma_{\mathrm{1u}} = 2.15 \pm 0.12$.

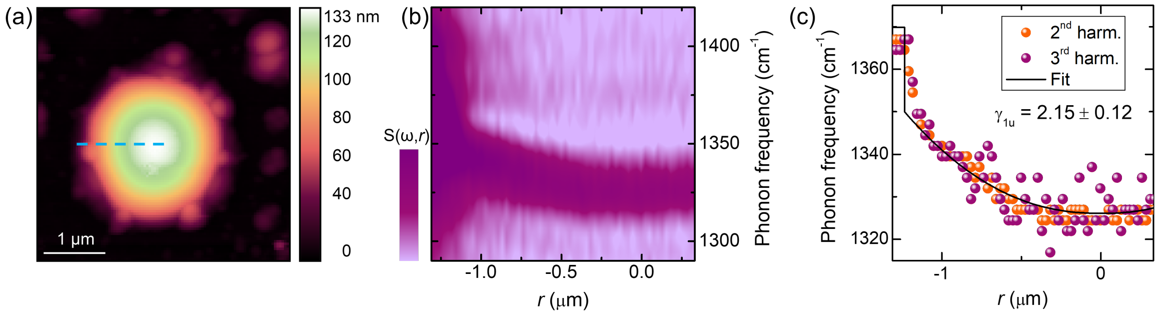

Figure 1.3: IR-active mode *vs* strain for a third bubble. (a) 2D AFM image of a circular hBN bubble, with $R = 1.23$ μm and $h_{\mathrm{m}} = 126$ nm ($h_{\mathrm{m}}/R = 0.102$) and created in an hydrogenated sample (beam energy equal to 34 eV). (b) Amplitude line scan of the 2nd harmonic signal associated to the IR active mode ($E_{1\mathrm{u}}$) along the cyan dashed line shown in panel (a). (c) IR phonon frequency dependence of the second and third harmonic line scans as a function of the radial distance r. The black solid line is a fit to the data assuming a linear dependence as extracted from the phonon frequency on $\varepsilon_{\mathrm{tot}}$, provided by Eqs. (3) and (5) of the main text.

Supporting Note 2. Raman mapping measurements in hBN bubbles

In this section, we show additional Raman mapping measurements taken along the diameter of other hydrogen- (or deuterium-) filled hBN bubbles. In panel (a) of Figs. 2.1 and 2.2, we show the false color plots of the intensity associated to the Raman spectra measured along the diameter of two different hBN bubbles. The most intense peak of the plots is assigned to the E_{2g} mode of the unstrained hBN bulk under the bubble. The weak peak that shifts as a function of the position of the excitation spot is the E_{2g} mode of the hBN layers comprised in the bubble. In panels (b), we show the corresponding Raman spectra, stacked by y-offset. Finally, in panels (c), we show the dependence of the Raman mode frequency as a function of the distance from the center r. We also show the fit to the experimental data to extract the Grüneisen parameter of the Raman mode.

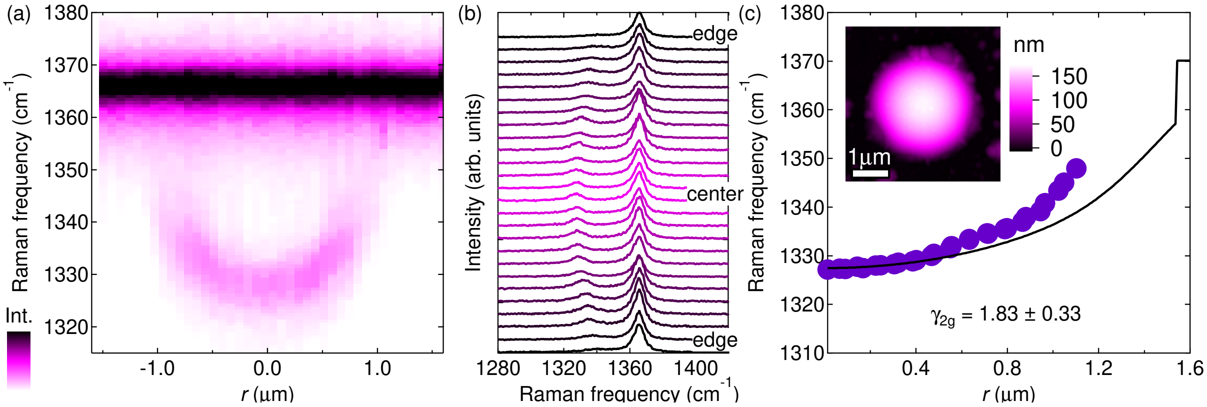

Figure 2.1: Summary of the Raman measurements performed on a hBN bubble with $R = 1.59$ μm and $h_m = 181$ nm ($h_m/R = 0.114$) and created in a deuterated sample (beam energy equal to 6 eV). (a) False color image of the intensity of Raman spectrum as a function of position along a diameter of a bubble. (b) Corresponding Raman spectra. (c) E_{2g} Raman mode frequencies as a function of the distance from the center of the bubble r. The solid line is a linear fit. The Grüneisen parameter is $\gamma_{2g} = 1.83 \pm 0.33$, which corresponds to a shift rate of $\Delta_{2g} = (25.1 \pm 4.5)$ cm^{-1}/%.

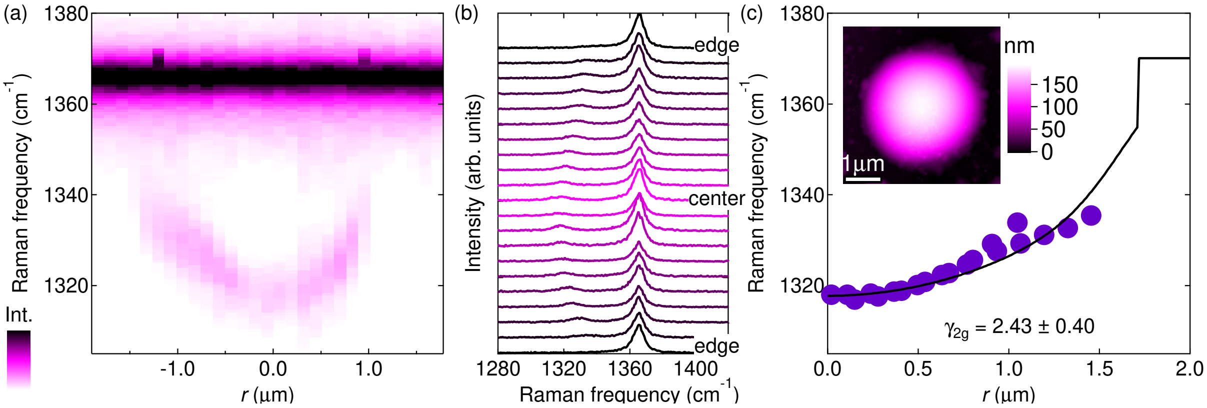

Figure 2.2: Summary of the Raman measurements performed on a hBN bubble with $R = 1.71$ μm and $h_m = 179$ nm ($h_m/R = 0.105$) and created in a deuterated sample (beam energy equal to 6 eV). (a) False color image of the intensity of Raman spectrum as a function of position along a diameter of a bubble. (b) Corresponding Raman spectra. (c) E_{2g} Raman mode frequencies as a function of the distance from the center of the bubble r. The solid line is a linear fit. The Grüneisen parameter found experimentally for this bubble is $\gamma_{2g} = 2.43 \pm 0.40$, which corresponds to a shift rate of $\Delta_{2g} = (33.2 \pm 5.2)$ cm^{-1}/%.

Supporting Note 3. Statistical analysis of the shift rates and Grüneisen parameters

To have a larger statistical analysis of the shift rates and Grüneisen parameters, we measured the Raman shifts at the center of about ten bubbles. We chose to perform Raman measurements rather than nano-FTIR measurements since the latter are more demanding. The resulting histogram is shown in Fig. 3.1. A Gaussian fit to the data provides an average shift with respect to the frequency of the unstrained membrane (assumed to be $\omega_{2g}^0 = 1370\ \mathrm{cm}^{-1}$) equal to 53 ± 11 cm^{-1}. To estimate the Grüneisen parameter, we need to estimate the average strain at the bubble center. Indeed, the latter is related to the aspect ratio via the following equation [2]:

$$\varepsilon^{\mathrm{center}} = 0.715 \cdot \left(\frac{h_{\mathrm{m}}}{R}\right)^2, \tag{3.3}$$

We thus considered the aspect ratios measured for a hundredth of bubbles (see Fig. 1(f) of the main text), and estimated a average value equal to 0.115 ± 0.011, see Fig. 3.1. In turn, from Eq. 3.3, we estimate $\varepsilon^{\mathrm{center}} = (1.90 \pm 0.35)\%$. We can finally estimate the average Grüneisen parameter as:

$$\gamma_{2\mathrm{g}} = \frac{\omega_{2\mathrm{g}}^{\mathrm{center}} - \omega_{2\mathrm{g}}^0}{\omega_{2\mathrm{g}}^0 \cdot \varepsilon^{\mathrm{center}}}, \tag{3.4}$$

getting $\gamma_{2\mathrm{g}} = 2.04 \pm 0.48$. This value agrees well with that estimated via Raman mapping measurements (see Fig. 3 of the main text and Supporting Note 2).

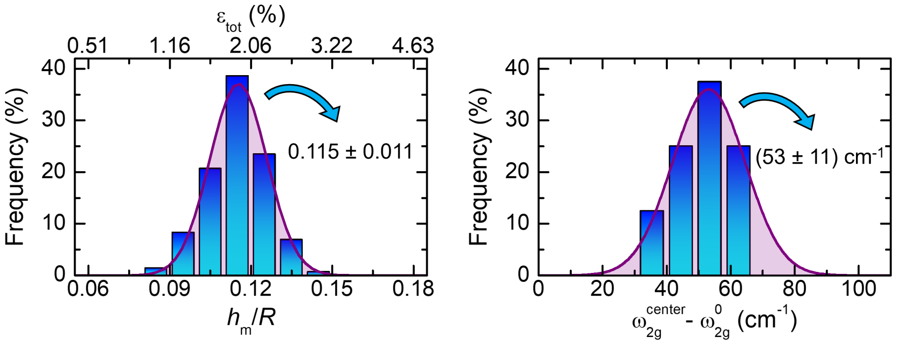

Figure 3.1: Histograms of the aspect ratios h_{m}/R (left) and Raman shifts measured at the summit of the bubbles $\omega_{2\mathrm{g}}^{\mathrm{center}} - \omega_{2\mathrm{g}}^0$ (right). The Raman shift was calculated with respect to the unstrained value, which is assumed to be $\omega_{2\mathrm{g}}^0 = 1370\ \mathrm{cm}^{-1}$. A Gaussian fit to the data (purple) provides the average values for both the two quantities.

Supporting Note 4. Polarization-resolved Raman measurements in hBN bubbles

Lineshape fitting of the polarization-resolved Raman measurements

To extract the intensity of the two Raman modes E_{2g}^{+} and E_{2g}^{-} as a function of the polarization angle of the Raman-scattered light ϕ, we performed a two-step analysis. We first fitted the spectra measured at different polarization angles with two Lorentzians (one for the Raman peak of the bubble, another for that of the unstrained bulk). This analysis allowed us to determine the maximum and minimum frequencies corresponding to the oscillating behavior observable in Fig. 5(a) of the main text. These maximum and minimum frequencies correspond to ω_{1u}^{+} and ω_{1u}^{-}, respectively. We then performed an additional fitting, using three Lorentzian functions, where we fixed the frequencies of the two Lorentzians used to fit the Raman peak of the bubble to the ω_{1u}^{+} and ω_{1u}^{-}. All the intensities shown in the main text and in the Supplementary Information are normalized to the intensity of the Raman peak of bulk hBN. E_{2g} modes of unstrained hBN are expected to show no dependence on the polarization of the scattered light [10], hence this normalization does not alter the polarization dependence of the $E_{2g}^{\pm}$ modes of the bubble.

In Fig. 4.1, we show the spectra measured at directions parallel ($\phi = 0°$) and perpendicular ($\phi = 90°$) with respect to the direction of the strain. The fitting of these two spectra demonstrate that the Raman peak measured on the bubble can be well reproduced with a single Lorentzian (the second Lorentzian with a negligible amplitude is also shown in Fig. 4.1). We show also the spectrum measured at an intermediate angle ($\phi = 135°$), in which the two Lorentzian curves the frequencies corresponding to the split Raman peaks have a comparable amplitude.

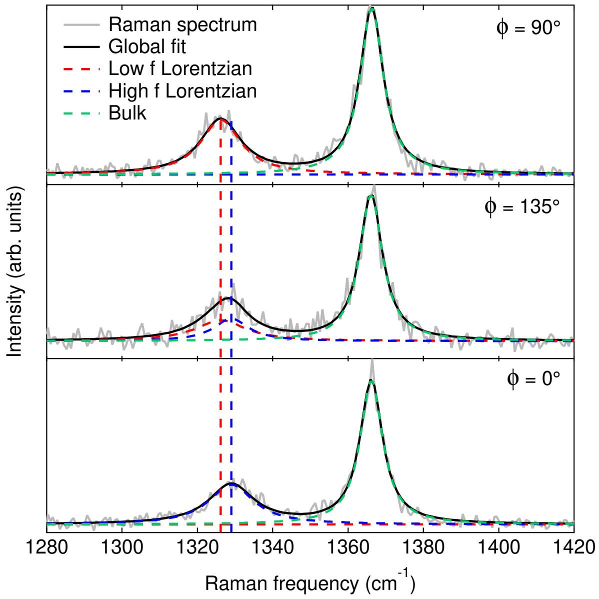

Figure 4.1: Raman spectra measured at three different polarization angles ϕ. In the bottom (top) panel, the spectrum measured at a polarization parallel (perpendicular) to the strain direction is shown. In the central panel, a spectrum measured at the intermediate angle is displayed.

Polarization measurements of another bubble

Analogous measurements to those shown in Fig. 5 of the main text were taken on a second bubble. The bubble has $R = 2.04$ μm and $h_m = 216$ nm ($h_m/R = 0.106$) and was created in a deuterated sample (beam energy equal to 25 eV). Fig. 4.2(a) shows an intensity map formed

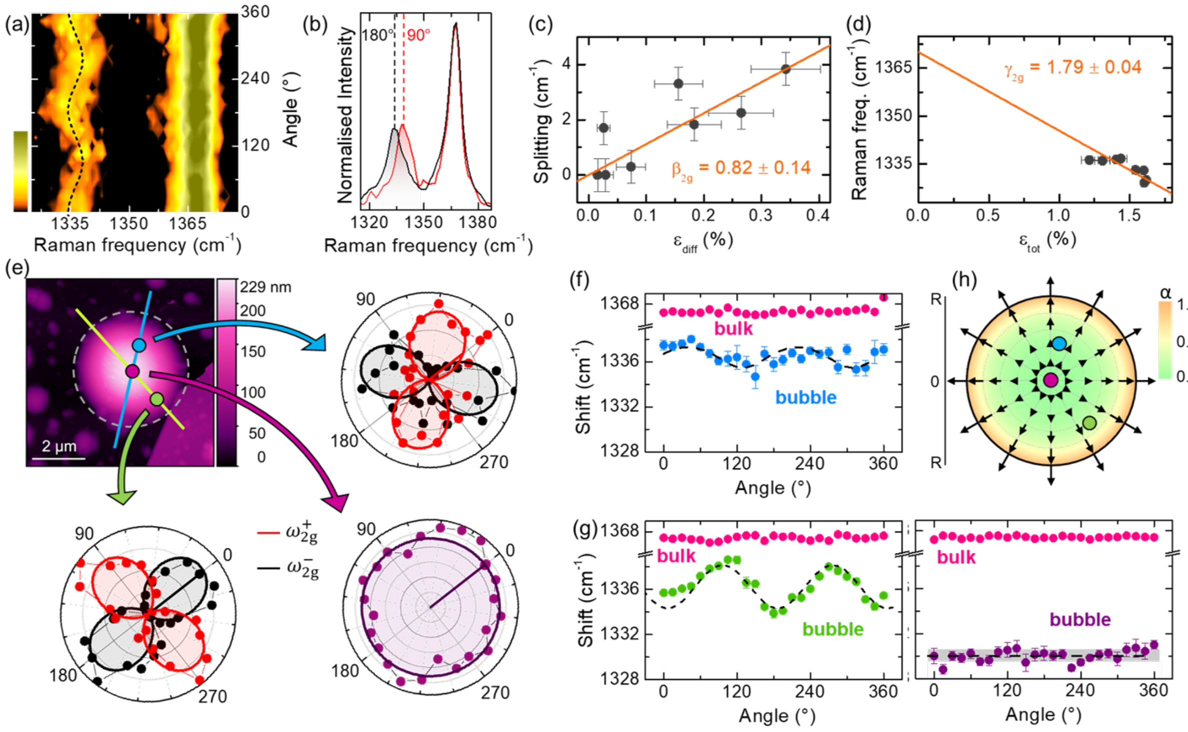

Figure 4.2: (a) Color map of the intensity of the E_{2g} Raman mode as a function of the angle of the polarization analyzer. The bulk mode at $\sim$1367 cm^{-1} shows no modulation. The dome mode at $\sim$1335 cm^{-1} shows a clear frequency oscillation, as highlighted by the black dashed line. (b) Two illustrative spectra corresponding to perpendicular angles equal to 90° and 180°. A splitting is clearly visible. (c-d) Summary of the splittings *vs* $\varepsilon_{\text{diff}}$ (c) and average frequencies *vs* ε_{tot} (d) measured by performing a similar analysis on several points over the bubble. The corresponding strain values were estimated via FEM calculations. The solid lines are linear fits. (e) AFM image of the studied hBN bubble. The measurements of panels (a-b) were acquired in the position highlighted by the green dot. Fitting procedures of the spectra allow us to derive the polar plot of the intensities of the low (black) and high (red) frequency Raman modes as a function of the analyzer angle. Analogous polar plots were derived from the measurements acquired on the purple and cyan dots. The solid lines in the polar plots are fits to the data. (f) Average frequency behavior obtained from the measurements acquired on the cyan dot. The dashed line follows the sinusoidal behavior of the frequency, which is caused by the strain anisotropy. (g) Average frequency behavior analogous to that of panel (f) but referring to the green and purple dots. (h) Spatial distribution of the strain anisotropy $\alpha = (\varepsilon_r - \varepsilon_\theta)/(\varepsilon_r + \varepsilon_\theta)$, determined based on FEM calculations. The arrows point to the direction of the strain field. Their length is calculated as $\log_{10}(100\alpha)$. The cyan, purple and green dots are in one-to-one correspondence with those superimposed to the AFM image of panel (e).

by polarization-dependent μ-Raman spectra recorded on a given point of the bubble. While the E_{2g} bulk mode at $\sim$ 1367 cm^{-1} remains constant in intensity and frequency, the strain-softened E_{2g} mode of the bubble at $\sim$ 1335 cm^{-1} exhibits a sinusoidal behavior of its center-of-mass frequency, pointing to a mode splitting. The splitting is clearly visible from the spectra of Fig. 4.2(b), recorded with opposite polarizations (ϕ = 180° and 90°, corresponding to a minimum and a maximum of the sinusoidal behavior, respectively). Similar measurements were taken on several other points of the bubble, in order to determine the splitting and average Raman frequency. From the knowledge of the radial distance r, it was possible to establish the radial and circumferential strain components corresponding to each set of data via FEM calculations. In panels (c) and (d), we display the measured splittings *vs* $\varepsilon_{\text{diff}}$ and the average frequencies *vs* ε_{tot}, respectively. From a linear fit, we could determine the shear deformation potential and Grüneisen parameter. From a detailed analysis of the polarization-resolved measurements, it is possible to determine also the angular dependence of the high-frequency and low-frequency modes. This is

exemplified in panel (e) for three different points of the bubble. The latter are highlighted by the colored dots superimposed to the AFM image of the bubble. It should be noticed that the data displayed in panels (a) and (b) were acquired on the green spot superimposed to the AFM image in panel (e). Indeed, the angular behavior follows the same rotation of the strain direction. To make it more apparent, the polar plots were rotated counter-clockwise by 38°. In such a manner, it is possible to notice how the red lobes (corresponding to the high-frequency mode) are always aligned parallel to the strain axes, indicated in cyan and green on the AFM image. At the bubble center (purple dot), instead, no splitting could be observed and the bubble mode remains constant in intensity when varying the angle of the polarization analyzer. Indeed, the existence of a splitting and in turn the presence of two oppositely-polarized modes is a consequence of the presence of an anisotropic strain. The extent of the anisotropy manifests itself in the amplitude of the oscillating behavior of the center of mass frequency. This is exemplified in panel (f) for the cyan point, where a splitting of 1.8 cm^{-1} was measured. Indeed, as shown in panel (g), an analysis of the center of mass frequency reveals a noticeably larger splitting for the green point —equal to 3.8 cm^{-1}— and the absence of splitting for the purple point. This is attributable to the fact the cyan point corresponds to an r value in between those of the purple point —where the anisotropy is null— and of the green point —where the anisotropy is larger— as highlighted by the anisotropy plot in panel (h).

Supporting Figure 1. Optical and AFM images of an H-irradiated flake

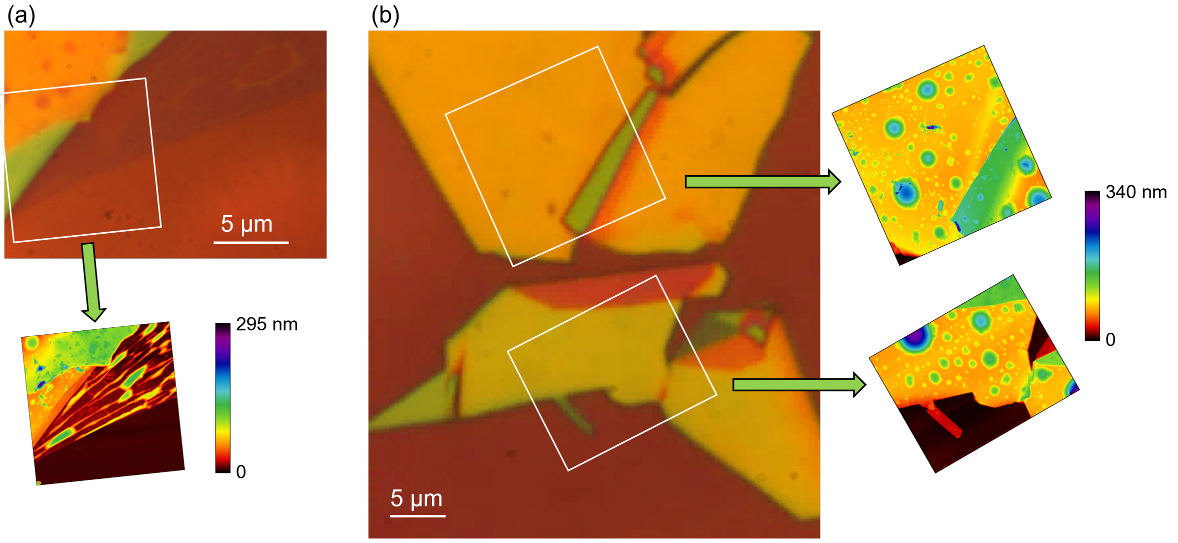

Figure S1. (a) Optical image of an H-irradiated hBN flake consisting of a thicker part on the left and a thinner part on the right. Elongated features can be barely seen in the thin part. AFM measurements confirm the presence of wrinkles and irregular structures. (b) Optical image of a thick H-irradiated hBN flake. Although optically the flake surface looks flat, AFM measurements reveal the presence of circular bubbles.

Supporting Figure 2. AFM characterization before and after H-irradiation

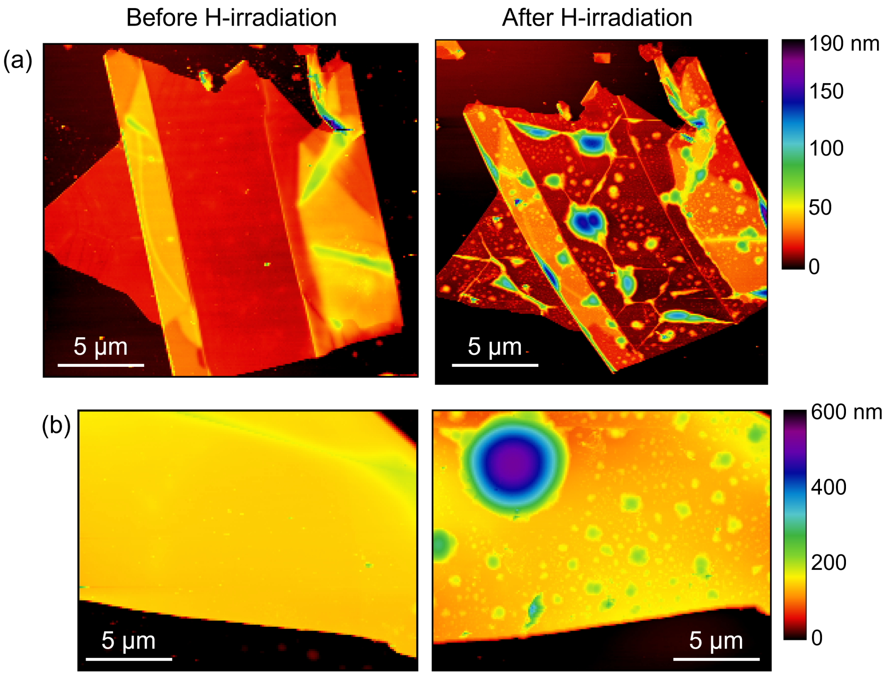

Figure S2. (a) AFM image of a hBN flake (thickness $t \approx 15$ nm) before and after H-irradiation. While prior to irradiation the flake surface looks flat, after the H-treatment bubbles, wrinkles and irregular structures are present on the flake. (b) Same for a thicker flake (thickness $t \approx 160$ nm). In this case, after the H-treatment only bubbles formed.

Supporting Figure 3. Bubbles and wrinkles in hBN

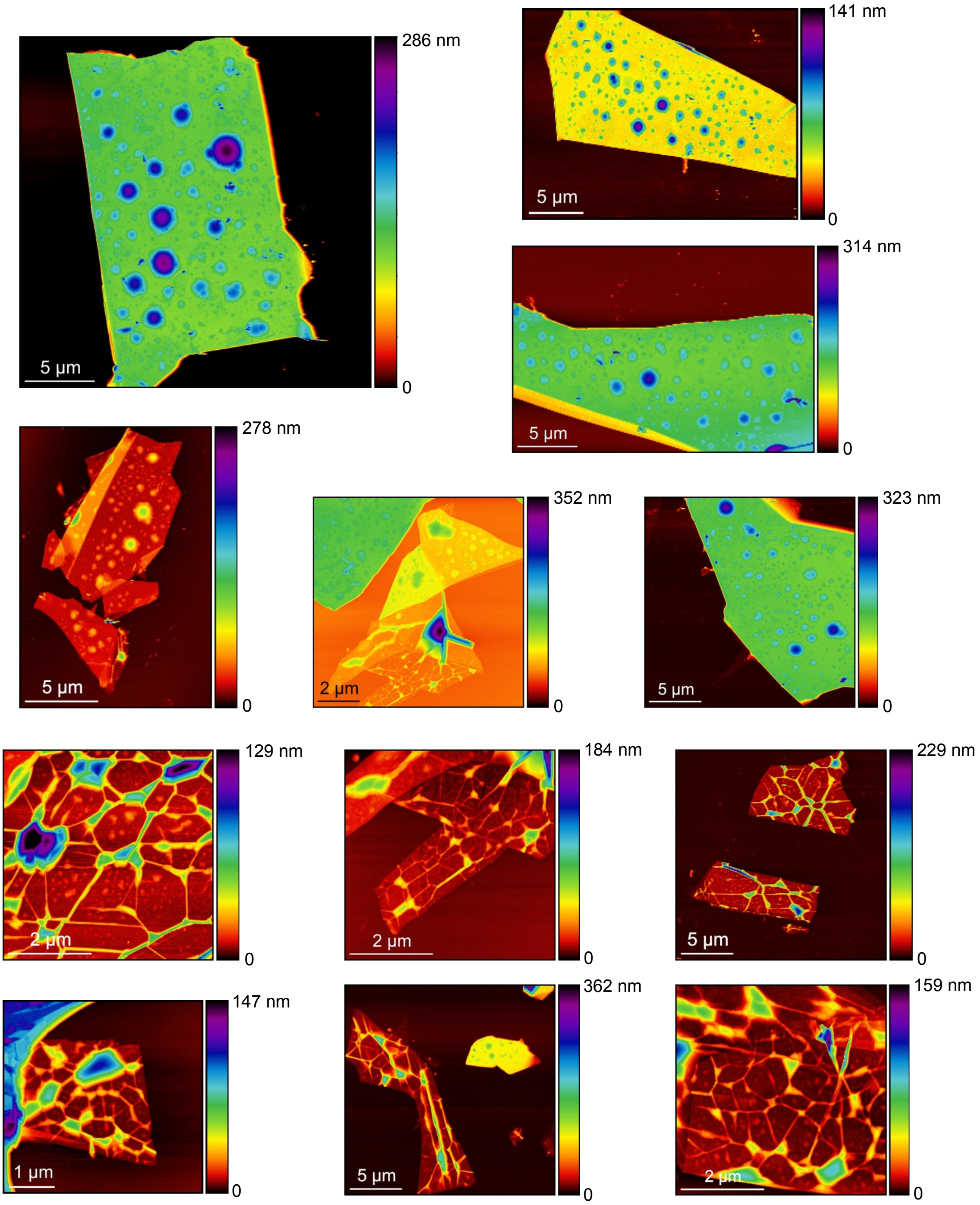

Figure S3. This figure shows AFM images of several hBN flakes following hydrogen- or deuterium- irradiation. As discussed in the main text, depending on the flake thickness either bubbles or wrinkles form.

The formation of wrinkles and irregular structures in the thinnest flakes ($t \lesssim 10\,\mathrm{nm}$) resembles the creation of irregularly shaped bubbles in graphene, achieved by high-energy ion-irradiation (500 keV) [11]. In that work, the density of observed features was much higher than the density of impinging ions (1×10^9 cm^{-2}), and the formation of these structures was thus attributed to an additional gas release from the SiO_2 substrate, with the gas remaining trapped

at the flake-substrate interface. The low beam energies used in our case rule out a significant gas release from the substrate [12]. However, here we employed ion doses 8 orders of magnitude larger than in Ref. [11], that justifies the formation of molecular hydrogen at the flake-substrate interface for $t \lesssim 10$ nm. The formed molecular hydrogen may accumulate and percolate, which originates irregular structures and wrinkles in the thinnest flakes (as shown in this figure and in Fig. 1(c) of the main text). On the other hand, the formation of spherically-shaped bubbles in thick flakes ($t \gtrsim 10$ nm) can be attributed to the formation of molecular hydrogen that remains trapped in the hBN interlayers, as observed in H plasma-treated hBN [13], and in TMD bubbles [14]. In this respect, one should consider that the bubbles form as a consequence of the energy balance between the gas expansion, the hBN membrane elastic energy and the adhesion energy between the hBN membrane and the parent flake beneath or the substrate [2]. The spherical shape of the bubbles derives from the minimization of the total energy of the system. Indeed, in between different crystal planes within a hBN flake, there is a perfect adhesion between the planes. This perfect adhesion guarantees that —when hydrogen molecules form— the gas expands isotropically, leading to the blistering of spherically-shaped bubbles. Instead, the SiO_2/hBN interface may be not as ideal due to a non-perfect adhesion between the flake and the substrate. This non-perfect adhesion can be caused by the deposition process itself, during which unavoidably present contaminants such as hydrocarbons or air are trapped, by the presence of debris, impurities or tape residuals, by the presence of steps in the deposited flake, by small strain transfers to the flake itself, etc. All these factors contribute to the creation of preferential directions along which the H_2 gas expands, giving rise to the formation of irregular structures and wrinkles.

Supporting Figure 4. Bubble thickness

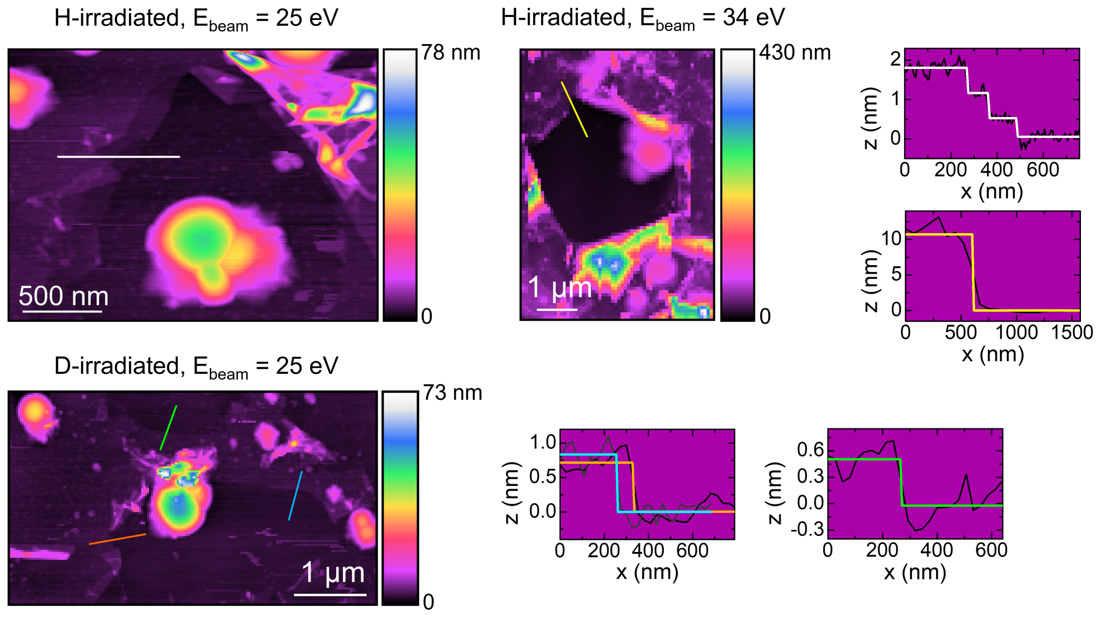

Figure S4. AFM images of three irradiated samples, after causing the explosion of some bubbles. The three samples were irradiated under different conditions, *i.e.* by using either hydrogen, H, or deuterium, D, ions and different beam energies.

In the sample irradiated with a 25-eV H-ion-beam (top left), the crater left by an exploded bubble can be seen. In one of the edges (white line) several steps can be noticed, suggesting that the bubble thickness was of several layers. By fitting the profile with a multiple step function (plot on the right), we estimate a total thickness of 1.8 nm. This crater is the shallowest one measured in hydrogenated samples. Interestingly, other bubbles are present within the crater, indicating how the exploded bubble possibly had a Russian-doll-like structure.

A crater can also be noticed in the sample irradiated with a 34-eV H-ion-beam (top center). In this case, it can be immediately noticed how the crater is deeper. A fit via a step function of the profile acquired along the yellow line points to a thickness of 10.7 nm. Other craters in hydrogenated samples featured thicknesses in between 2 and 12 nm.

Finally, in the sample deuterated with a 25-eV D-ion-beam, two craters can be noticed in the top part and bottom part of the image. One of the craters (bottom one) is relatively big, and by analysing two profiles — acquired along the orange and cyan lines — we estimated thicknesses of 0.71 nm (orange profile) and 0.83 nm (cyan one). The smaller crater in the top part of the image shows an ever smaller thickness of 0.52 nm, as estimated by an analysis of the profile acquired along the green line.

We measured several other profiles and our analysis suggest that in deuterated samples (beam energies between 6 eV and 25 eV) the bubbles are generally thinner than those formed in hydrogenated samples (beam energies between 6 eV and 34 eV).

Supporting Figure 5. Durability of hBN bubbles

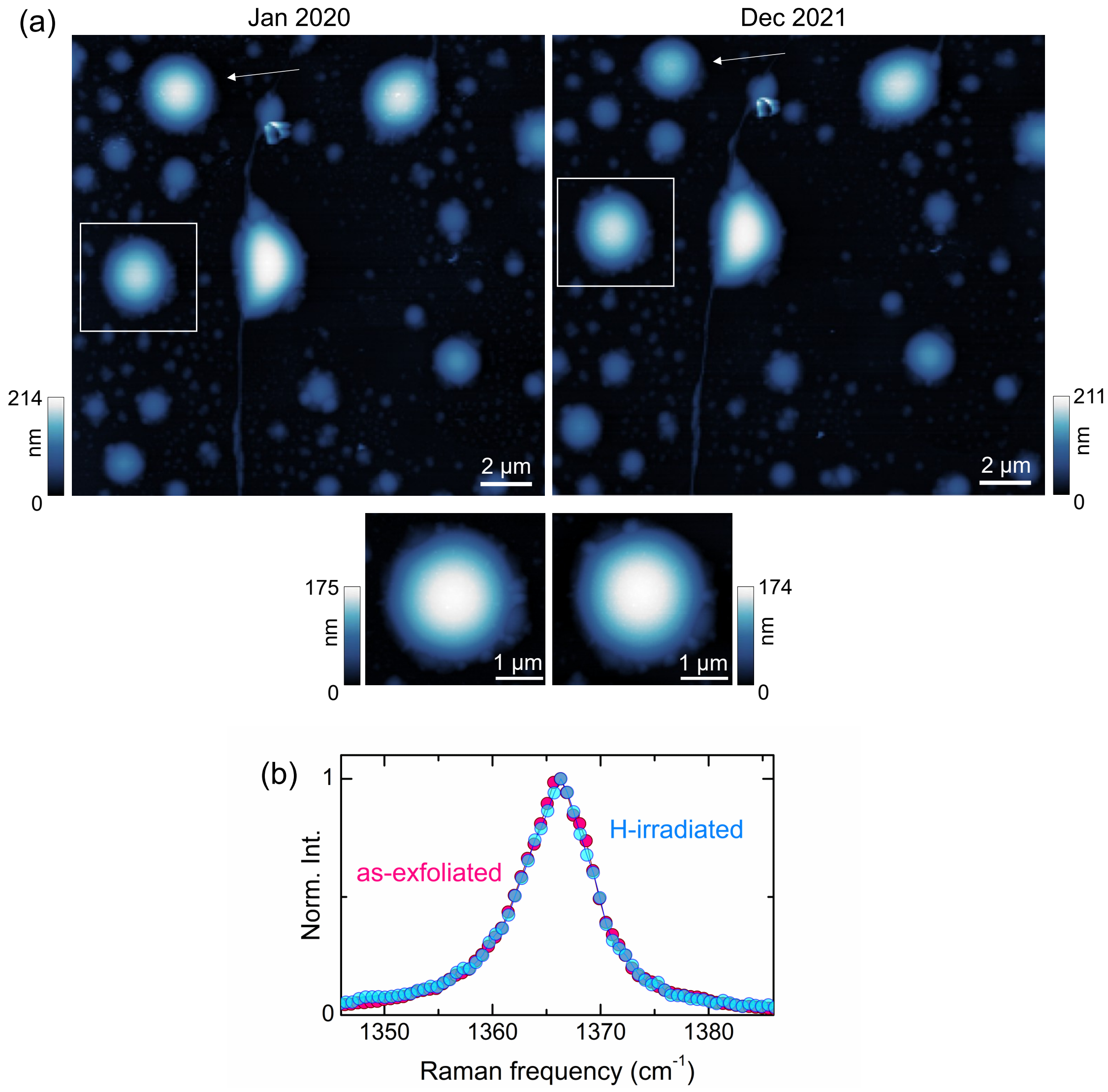

Figure S5. (a) Top panels: AFM images of an hBN flake irradiated with a 6-eV deuterium beam. The image on the right was recorded 23 months after that on the left, in about the same area. The white arrows indicate a bubble, which deflated over time. The other bubbles remained instead almost unchanged. In particular, the white squares highlight a bubble, whose higher resolution AFM images are displayed in the bottom panels. Bottom panels: Higher-resolution AFM images of a same bubble recorded after 23 months, showing that no sizable variations in the bubble size and shape occurred after about two years. (b) μ-Raman spectra of the E_{2g} hBN bulk mode recorded on an as-exfoliated (magenta dots) and H-irradiated (light blue dots). The latter was acquired on a flat area close to a bubble. No variation in the mode lineshape can be noticed after H irradiation.

Supporting Figure 6. AFM and FEM calculations of the bubble studied by Raman spectroscopy

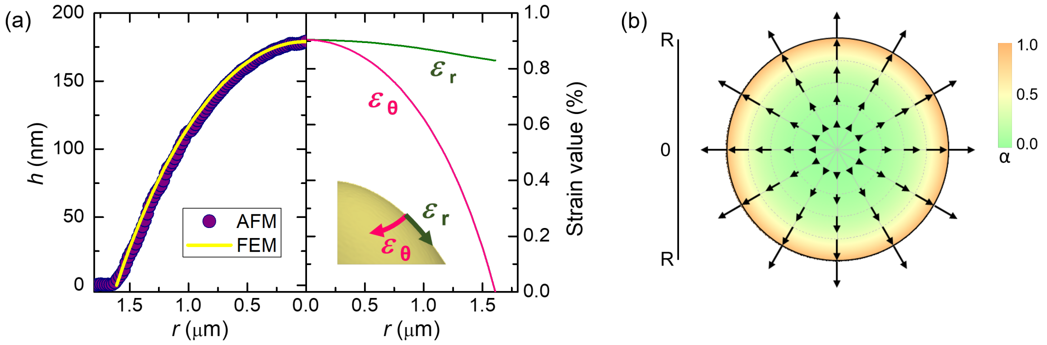

Figure S6. (a) Left: AFM profile (circles) along a radius of the bubble studied by Raman spectroscopy (see Figs. 4 and 5 of the main text). The yellow line is the height profile simulated via FEM numerical calculations. Right: Radial dependence —obtained by FEM calculations— of the in-plane circumferential (ε_θ) and radial (ε_r) strain components, a sketch of which is depicted as inset. (b) Spatial distribution of the strain anisotropy, defined as: $\alpha = (\varepsilon_r - \varepsilon_\theta)/(\varepsilon_r + \varepsilon_\theta)$. The arrows indicate the direction of the strain field —dictated by the radial strain component— and their length is proportional to $\log_{10}(100 \cdot \alpha)$.

References

[1] P. Wang, W. Gao, Z. Cao, K. M. Liechti, and R. Huang, *Numerical analysis of circular graphene bubbles*, J. Appl. Mech. **80**, 040905 (2013).

[2] E. Blundo, T. Yildirim, G. Pettinari, and A. Polimeni, *Experimental Adhesion Energy in van der Waals Crystals and Heterostructures from Atomically Thin Bubbles*, Phys. Rev. Lett. **127**, 4, 046101 (2021).

[3] F. Huth, A. Govyadinov, S. Amarie, W. Nuansing, F. Keilmann, and R. Hillenbrand, *Nano-FTIR absorption spectroscopy of molecular fingerprints at 20 nm spatial resolution*, Nano Lett. **12**, 8, 3973–3978 (2012).

[4] S. Mastel, A. A. Govyadinov, T. V. de Oliveira, I. Amenabar, and R. Hillenbrand, *Nanoscale-resolved chemical identification of thin organic films using infrared near-field spectroscopy and standard Fourier transform infrared references*, Appl. Phys. Lett. **106**, 2, 023113 (2015).

[5] Z. Shi, H. A. Bechtel, S. Berweger, Y. Sun, B. Zeng, C. Jin, H. Chang, M. C. Martin, M. B. Raschke, and F. Wang, *Amplitude- and phase-resolved nanospectral imaging of phonon polaritons in hexagonal boron nitride*, ACS Photonics **2**, 7, 790–796 (2015).

[6] P. Li, M. Lewin, A. V. Kretinin, J. D. Caldwell, K. S. Novoselov, T. Taniguchi, K. Watanabe, F. Gaussmann, and T. Taubner, *Hyperbolic phonon-polaritons in boron nitride for near-field optical imaging and focusing*, Nat. Commun. **6**, 1, 7507 (2015).

[7] P. Schmidt, F. Vialla, S. Latini, M. Massicotte, K.-J. Tielrooij, S. Mastel, G. Navickaite, M. Danovich, D. A. Ruiz-Tijerina, C. Yelgel, V. Fal'ko, K. S. Thygesen, R. Hillenbrand, and F. H. L. Koppens, *Nano-imaging of intersubband transitions in van der Waals quantum wells*, Nat. Nanotech. **13**, 1035 (2018).

[8] S. L. Moore, C. J. Ciccarino, D. Halbertal, L. J. McGilly, N. R. Finney, K. Yao, Y. Shao, G. Ni, A. Sternbach, E. J. Telford, B. S. Kim, S. E. Rossi, K. Watanabe, T. Taniguchi, A. N. Pasupathy, C. R. Dean, J. Hone, P. J. Schuck, P. Narang, and D. N. Basov, *Nanoscale lattice dynamics in hexagonal boron nitride moiré superlattices*, Nat. Commun. **12**, 5741 (2021).

[9] A. A. Govyadinov, S. Mastel, F. G. F, A. Chuvilin, P. S. Carney, and R. Hillenbrand, *Recovery of permittivity and depth from near-field data as a step toward infrared nanotomography*, ACS Nano **8**, 6911 (2014).

[10] Y. Wang, C. Cong, C. Qiu, and T. Yu, *Raman spectroscopy study of lattice vibration and crystallographic orientation of monolayer MoS_2 under uniaxial strain*, Small **9**, 17, 2857–2861 (2013).

[11] E. Stolyarova, D. Stolyarov, K. Bolotin, S. Ryu, L. Liu, K. T. Rim, M. Klima, M. Hybertsen, I. Pogorelsky, I. Pavlishin, K. Kusche, J. Hone, P. Kim, H. L. Stormer, V. Yakimenko, and G. Flynn, *Observation of graphene bubbles and effective mass transport under graphene films*, Nano Lett. **9**, 332 (2009).

[12] A.-M. Lanzillotto, T. E. Madey, and R. A. Baragiola, *Negative-ion desorption from insulators by electron excitation of core levels*, Phys. Rev. Lett. **67**, 2, 232 (1991).

[13] L. He, H. Wang, L. Chen, X. Wang, H. Xie, C. Jiang, C. Li, K. Elibol, J. Meyer, K. Watanabe, T. Taniguchi, Z. Wu, W. Wang, Z. Ni, X. Miao, C. Zhang, D. Zhang, H. Wang, and X. Xie, *Isolating hydrogen in hexagonal boron nitride bubbles by a plasma treatment*, Nat. Commun. **10**, 2851 (2019).

[14] D. Tedeschi, E. Blundo, M. Felici, G. Pettinari, B. Liu, T. Yildrim, E. Petroni, C. Zhang, Y. Zhu, S. Sennato, Y. Lu, and A. Polimeni, *Controlled Micro/Nanodome Formation in Proton-Irradiated Bulk Transition-Metal Dichalcogenides*, Adv. Mater. **31**, 1903795 (2019).
